# The Evolving Proteome of a Complex Extracellular Matrix, the *Oikopleura* House

**DOI:** 10.1371/journal.pone.0040172

**Published:** 2012-07-05

**Authors:** Julia Hosp, Yoshimasa Sagane, Gemma Danks, Eric M. Thompson

**Affiliations:** 1 Sars International Centre for Marine Molecular Biology, University of Bergen, Bergen, Norway; 2 Computational Biology Unit, University of Bergen, Bergen, Norway; 3 Department of Biology, University of Bergen, Bergen, Norway; Ecole Normale Supérieure de Lyon, France

## Abstract

Extracellular matrices regulate biological processes at the level of cells, tissues, and in some cases, entire multicellular organisms. The subphylum Urochordata exemplifies the latter case, where animals are partially or completely enclosed in “houses” or “tunics”. Despite this common strategy, we show that the house proteome of the appendicularian, *Oikopleura*, has very little in common with the proteome of the sister class, ascidian, *Ciona*. Of 80 identified house proteins (oikosins), ∼half lack domain modules or similarity to known proteins, suggesting *de novo* appearance in appendicularians. Gene duplication has been important in generating almost 1/3 of the current oikosin complement, with serial duplications up to 8 paralogs in one family. Expression pattern analyses revealed that individual oikosins are produced from specific fields of cells within the secretory epithelium, but in some cases, migrate up to at least 20 cell diameters in extracellular space to combine in defined house structures. Interestingly, peroxidasin and secretory phospholipase A_2_ domains, implicated in innate immune defence are secreted from the anlage associated with the food-concentrating filter, suggesting that this extra-organismal structure may play, in part, such a role in *Oikopleura*. We also show that sulfation of proteoglycans is required for the hydration and inflation of pre-house rudiments into functional houses. Though correct proportioning in the production of oikosins would seem important in repetitive assembly of the complex house structure, the genomic organization of oikosin loci appears incompatible with common enhancers or locus control regions exerting such a coordinate regulatory role. Thus, though all tunicates employ extracellular matrices based on a cellulose scaffold as a defining feature of the subphylum, they have evolved radically different protein compositions associated with this common underlying structural theme.

## Introduction

Cellulose produced by plants, bacteria and some fungi, is the most abundant biopolymer on earth. Animals are incapable of cellulose synthesis, with one exception, marine tunicates. Tunicates are the closest relatives to vertebrates, and develop a notochord and dorsal nerve cord during their larval stages. Among the three tunicate sister classes, Appendicularians, or Larvaceans, retain the larval appearance throughout their life cycle and remain pelagic, in contrast to benthic ascidians. They secrete a complex extracellular filter-feeding house, which in the Oikopleuridae completely surrounds the animal. The house consists of several chambers and filter sets made of cellulose and protein [1], [2]. Houses are regularly discarded and re-synthesized by the animals and abandoned houses are an important contribution to global vertical carbon flux [3]. The ability of tunicates to synthesise cellulose has most probably evolved after a horizontal gene transfer event, as the tunicate cellulose synthase (CesA) is more closely related to prokaryotic enzymes than plant CesA [4], [5]. Appendicularians possess two CesA genes with distinct temporal and functional specializations during larval tail development and post-metamorphic house synthesis [5], as opposed to sister ascidians with only one cellulose synthase gene.

**Figure 1:**
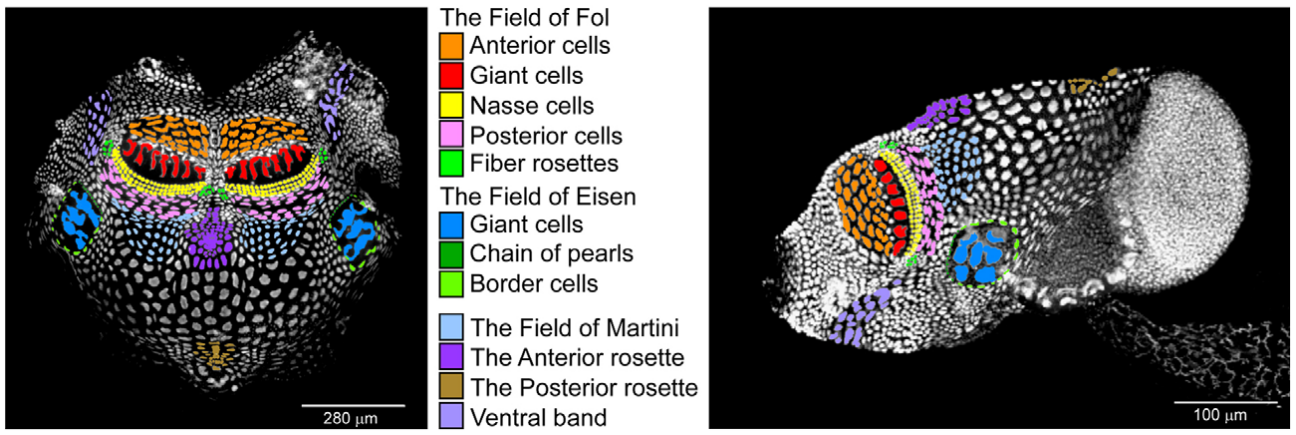
Cellular fields in the oikoplastic epithelium of Oikopleura *dioica*. Left, dorsal view where the epithelium of a day 6 animal has been slit ventrally, isolated from the animal and spread on a slide, anterior at top. Right, lateral view of a whole mount day 4 animal, mouth to the left, gonad at right, tail projecting to bottom right. Nuclei were stained with Hoechst (left image) and To-Pro3 (right image). Specific epithelial cellular fields are indicated by arbitrarily colored nuclei according to the central legend.

*Oikopleura dioica,* the only known dioeceous tunicate, can be cultured year round with a maturation time of 6 days at 15°C [6]. The trunk of the animal is surrounded by a highly polyploid oikoplastic epithelium of about 2000 cells [7]. This monolayer epithelium can be divided into distinct territories based on varying ploidy levels, shapes and nuclear morphologies of cells. Among these territories the Fol region is responsible for secretion of the food-concentrating filter (*fcf*) and the Eisen region produces the inlet filter (*if*) (see **Fig. 1** and [8]). House production initiates as the secretion of a compressed, pre-house rudiment directly above the epithelium. Following initial hydration and swelling of the pre-house rudiment in the dorsal-posterior region, houses are then inflated by specific tail movements and once fully expanded, sinusoidal movements of the tail control water flow though the filter chambers. Seawater enters the house through the *if*, where large particles are sieved out and then passes through the *fcf*, where appropriately sized particles are trapped and brought to the mouth where they are ingested with the aid of a mesh secreted by the endostyle.

In plants, cortical microtubules guide cellulose microfibril deposition from moving CesA complexes [9], whereas in *Oikopleura*, cellulose is secreted from stationary enzyme complexes, and a cortical F-actin array acts in part to guide microfibril deposition [8]. Microtubules appear to be involved in maintaining CesA complexes at the membrane and transport of house protein components via the Golgi network is important for correct house structure. Seven highly glycosylated house proteins (oikosins) identified using proteomic and Representation Difference Analysis (RDA) approaches are expressed in specific sub-regions of the epithelium [10], [11]. Similarities to known proteins were restricted to domains similar to vertebrate mucin sequences and cartilage intermediate protein (CLIP) in oikosin 1 (oik1) and the CUB domains found in known extracellular proteins in oik4 and 6 [12]. Oik1 is exclusively expressed in the seven giant Fol cells while oik3 and 5 are restricted to the anterior Fol area. The other oikosins have more complex spatial expression patterns. Immunolocalisation studies revealed that oikosins are indeed secreted into the filter structures, sometimes colocalising with and coating cellulose microfibrils [8]. To obtain a more comprehensive view of the complement and origins of proteins involved in the construction of the oikopleurid house, we expanded the proteomic analysis and have now characterized 66 new oikosin proteins expressed in distinct epithelial regions.

## Results and Discussion

### Secretion of Oikosins

The protein mass spectrometric analysis of pre-house rudiments performed in this study identified 66 new oikosin proteins (accession numbers: **Data S1**) to add to the complement of 14 oikosins that had been previously characterized [8], [10], [11]. As expected, since they were isolated from extracellular, pre-house rudiments, oikosins were generally classified as secreted proteins. The N-terminal of most oikosins (59/80) began with an M(K/R)(I/L/F) tripeptide signature. Using SignalP 4.0, 18 oikosins score strong predictions for a signal peptide (**Table S1**). SecretomeP 2.0, which also takes into account non-classical secretion predicts an additional 50 oikosins to be secreted (**Table S2**). Increasing numbers of soluble secretory proteins lacking conventional signal peptides are being reported. They circumvent the prevailing vesicular transport pathway and are secreted in a non-classical manner by a combination of four hypothetical pathways: secretion by endolysosomes, exosomes, membrane blebbing or transporters [13]–[15]. Only 12 oikosins (oik13, 17a, 21a,b, 22, 23, 30e, 33a, 34a,b, 35, and 43) lack predictions for secretion by either the signal peptide pathway or through non-classical secretion. This is despite their isolation from an extracellular matrix, the presence of numerous glycosylation sites and the presence of extracellular protein domains typical of secreted proteins.

**Figure 2:**
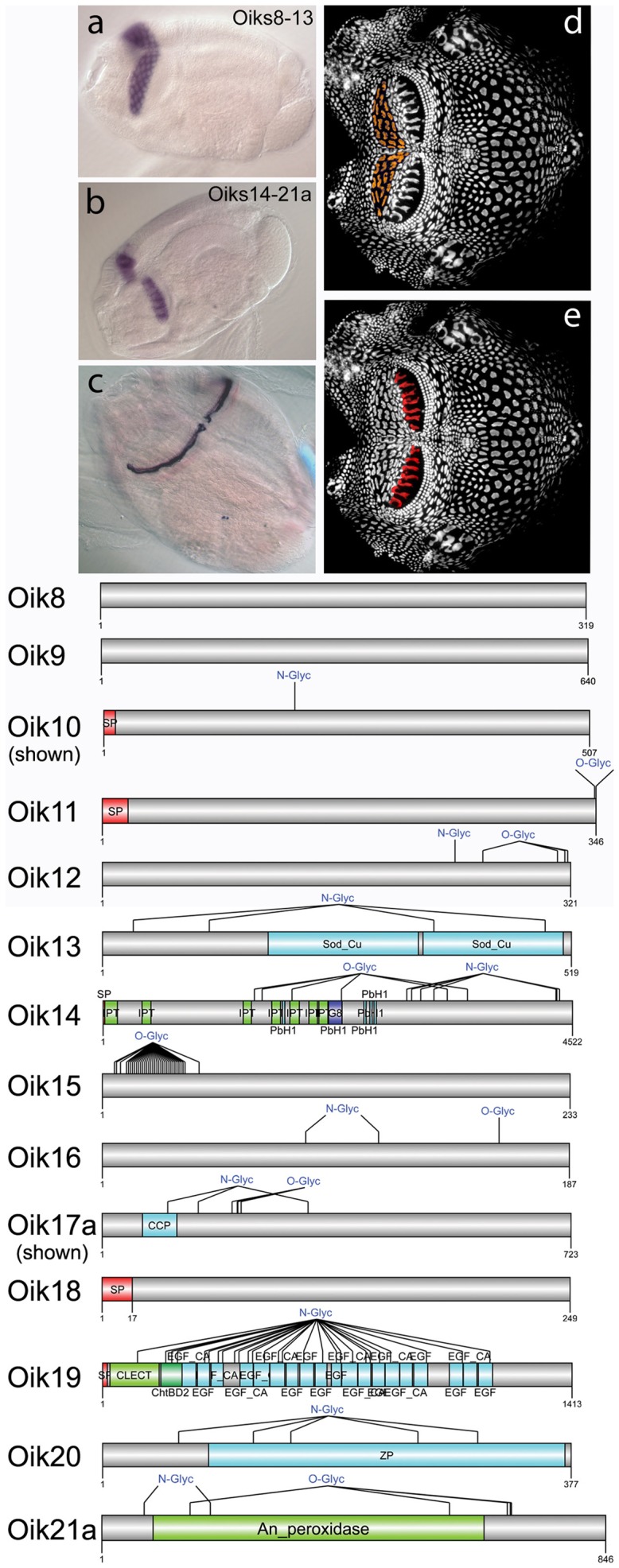
Oikosins expressed in anterior and giant Fol cells. (a,d) Oikosins 8–13 are expressed only in the anterior Fol. The *in situ* pattern for oik10 is shown in a, and the corresponding oikoplastic epithelial region indicated with orange coloring in d (anterior to left). *In situ* stains for the other oikosins exhibiting this pattern are provided in **Fig. S1**. (b,e) Oikosins 14–21 are expressed only in the giant Fol. The *in situ* pattern for oik17a is shown in b, and the corresponding oikoplastic epithelial region indicated with red coloring in e. *In situ* stains for the other oikosins exhibiting this pattern are provided in **Fig. S2**. (c) Sense control probe hybridizations for a number of oikosin mRNAs (and other unrelated mRNAs) exhibited the staining shown in this panel. This extracellular, non-specific “sticky” region on pre-house rudiments was systematically excluded in the assessment of all staining patterns in this study. Protein domain and modification schemas are shown below for oikosins 8–21: Sp, signal peptide; N- or O-Glyc, predicted N and O glycosylation sites; Sod_Cu, Copper/zinc superoxide dismutase domain; IPT, immunoglobulin-plexin-transcription domain of plexins and cell surface receptors, G8, domain containing 8 conserved glycines; PbH1, parallel beta-helix repeats, CCP, complement control protein modules, also known as short consensus repeats SCRs or SUSHI repeats; CLECT, c-lectin domain; ChtBD2, chitin binding domain; EGF, epidermal growth factor domain; EGF_CA, calcium binding epidermal growth factor-like domain; ZP, zona pellucida domain; An-peroxidase, peroxinectin_like animal heme peroxidase domain. *In situ* images are oriented with the oral cavity towards the left and were performed on day 3 animals with trunk lengths ranging from 350–400 µm in size.

### Oikosins are Expressed in Distinct Epithelial Regions of Post-metamorphic *Oikopleura* and Some have Modular Domain Organizations

All newly identified oikosins exhibited specific epithelial *in situ* expression patterns. In general, oikosins lack any sequence similarities to known proteins outside of known domain modules (see below). Almost half (32 of 80) contain no known protein domains at all. Among oikosins expressed in the anterior Fol region (**Fig. 2**), only oik13 contains known domains, namely two superoxide dismutase domains in its C-terminal half. In addition to the previously identified oik1 [10] a further eight oikosins were found to be expressed in the giant Fol cells (**Fig. 2**). Oik14 exhibits overall similarity to fibrocystin/polyductin (FPC), a kidney protein involved in fluid sensation and mechanotransduction [16]. The short, C-terminal transmembrane region of FPC might be absent in oik14, as indicated by a very low probability score (<0.2 for oik14 in contrast to >0.9 for FPC). Similar to FPC, however, oik14 contains several IPT/TIG domains, sequence stretches with an immunoglobulin-like fold that are found in cell surface receptors involved in the control of cell dissociation, motility, and invasion of extracellular matrices. Oik17a contains a CCP module [17] that is widespread in proteins of the complement system, but also found in many other proteins of diverse function, including those involved in cell adhesion. Oik19, a fibrillin-1 homolog containing multiple characteristic EGF-like domains, a chitin binding domain and several c-lectin domains, is also expressed in giant Fol cells. Lectins are non-enzymatic proteins present in plants and animals, which specifically bind to carbohydrate structures, such as cellulose scaffolds present in the *fcf* [5], [8], and play an important role in cell recognition. A zona pellucida (ZP) domain reported to act as a polymerization domain and to form supramolecular filaments [18] was found in oik20. Both fibrillin and ZP domains might be involved in stabilization of the cellulose threads in the *fcf* and/or construction of mesh-like elements within the cellulose scaffold. Oik21a,b, each containing a peroxidase domain, show overall similarity to peroxidasin which acts as an extracellular factor stabilizing the extracellular matrix (ECM) by cross-linking component proteins such as collagen and laminin [19]. This domain is also found in peroxinectin, an arthropod protein implicated in invertebrate immunity mechanisms [20] where it acts as a cell adhesive and opsonic peroxidase.

**Figure 3:**
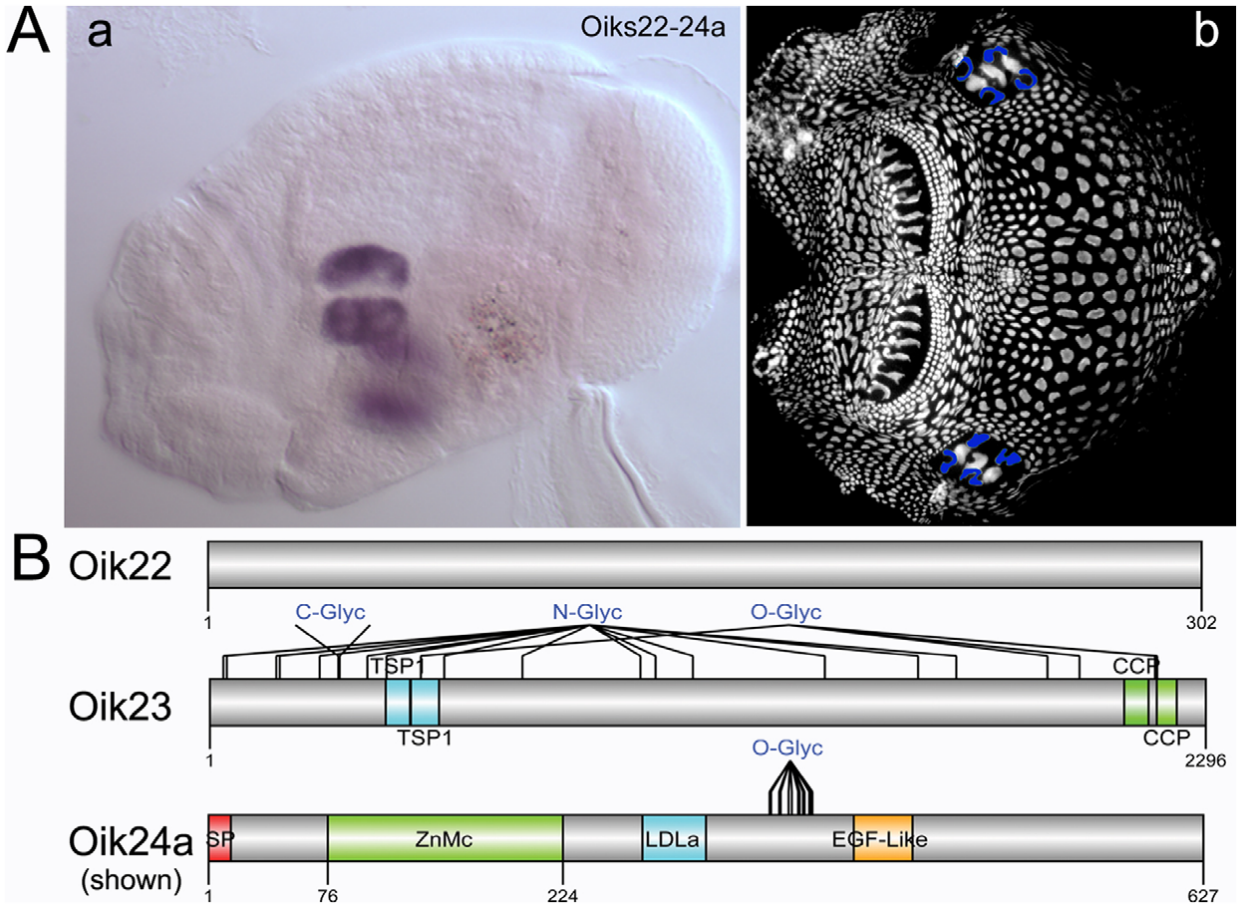
Oikosins expressed in the four lateral giant Eisen cells. (a,b) Oikosins 22–24 are expressed only in the four lateral giant Eisen cells. The *in situ* pattern for Oikosin 24a is shown in a, and the corresponding oikoplastic epithelial region indicated with blue coloring in b (anterior to left). *In situ* stains for the other oikosins exhibiting this pattern are provided in **Fig. S3**. Protein domain and modification schemas are shown below for oikosins 22–24: Sp, signal peptide; C-, N- or O-Glyc, predicted C, N and O glycosylation sites; TSP1, thrombospondin domain; CCP, complement control protein modules, also known as short consensus repeats SCRs or SUSHI repeats; ZnMc, Zinc-dependent metalloprotease domain; LDLa, Low-density lipoprotein receptor domain class A; EGF-Like: epidermal growth factor-like domain. *In situ* image is oriented with the oral cavity towards the left and was performed on a day 3 animal with trunk length of 380 µm.

Three different oikosin families were detected in the giant Eisen region (**Fig. 3**), one of which contains 8 paralogs, oik24a-h (**Fig. S4**), which together with the chain of pearls constitute the area of *if* secretion. Expression was restricted to the 4 lateral giant cells. Thus far only oik2 is found to be expressed in the three central giant Eisen cells, though it is not exclusive to these cells and is expressed elsewhere in the epithelium [10]. Oik24 paralogs are similar in amino acid sequence and domain structure. Their metalloproteinase domains have sequence similarity to extracellular Zn-dependent metalloproteinases of the astacin type. In addition to an EGF-like domain, the proteins also contain a low-density lipoprotein receptor class A domain (LDLa), the binding site for LDL and calcium within the LDL receptor that plays a central role in mammalian cholesterol metabolism [21]. Many predicted glycosylation sites are present within oik23 which also contains two thrombospondin repeats and two CCP domains. Thrombospondins (TSPs) are multimeric multidomain glycoproteins that function at cell surfaces and in the extracellular matrix. TSP repeats are believed to be involved in cell-cell interaction, inhibition of angiogenesis and apoptosis [22], [23].

**Figure 4:**
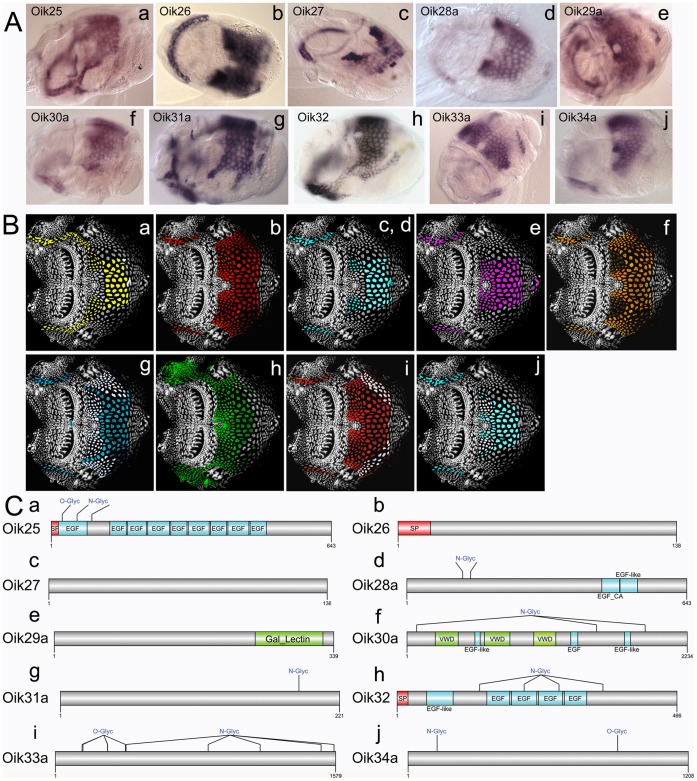
Oikosins expressed in dorsal domains with lateroventral projections. (A) *In situ* hybridisation patterns for oikosins: a) oik25, b) oik26, c) oik27, d) oik28a, e) oik29a, f) oik30a, g) oik31a, h) oik32, i) oik33a, and j) oik34a. B) Oikosin expression patterns in A are indicated by corresponding coloured domains on epithelial spreads (anterior to left). C) Protein domain and modification schemas are shown for oikosins 25–34: Sp, signal peptide; N- or O-Glyc, predicted N and O glycosylation sites; EGF, epidermal growth factor domain; EGF_CA, calcium binding epidermal growth factor-like domain; Gal_Lectin, galactose binding lectin domain; VWD, von Willebrand factor type D domain. *In situ* images are oriented with the oral cavity towards the left and were performed on day 3 animals with trunk lengths ranging from 350–400 µm in size.

A number of oikosins showed saddle-like expression patterns with transcripts in the dorsal posterior area, where initial prehouse swelling immediately precedes full expansion of a new house, and extensions in a ventral band (**Fig. 4**). This group is particularly rich in gene duplications as five of the families (oik28, 29, 31, 33, and 34) have 2 paralogs and one (oik30) has 5 paralogs. Oikosins 25, 28, and 32 contain a number of EGF and EGF_Ca domains that are found frequently in extracellular proteins, the latter thought to be important in numerous protein-protein interactions. Oik30a-e exhibit overall similarity to alpha-tectorin, one of the major noncollagenous compounds of the extracellular matrix of the inner ear that forms the tectorial membrane. Oik30 paralogs, however, do not contain a ZP domain typical of alpha-tectorins but do contain von Willebrand factor (VWF) D domains (VWD). In VWF the VWD are critical for multimizeration of VWF dimers [24], and indicate that mediation of protein-protein interactions may be a significant role of the oik30 family. The presence of a C-lectin domain in oik30e (**Fig. S4**) suggests that part of this mediation may also involve interactions with the cellulose scaffold of the house.

**Figure 5:**
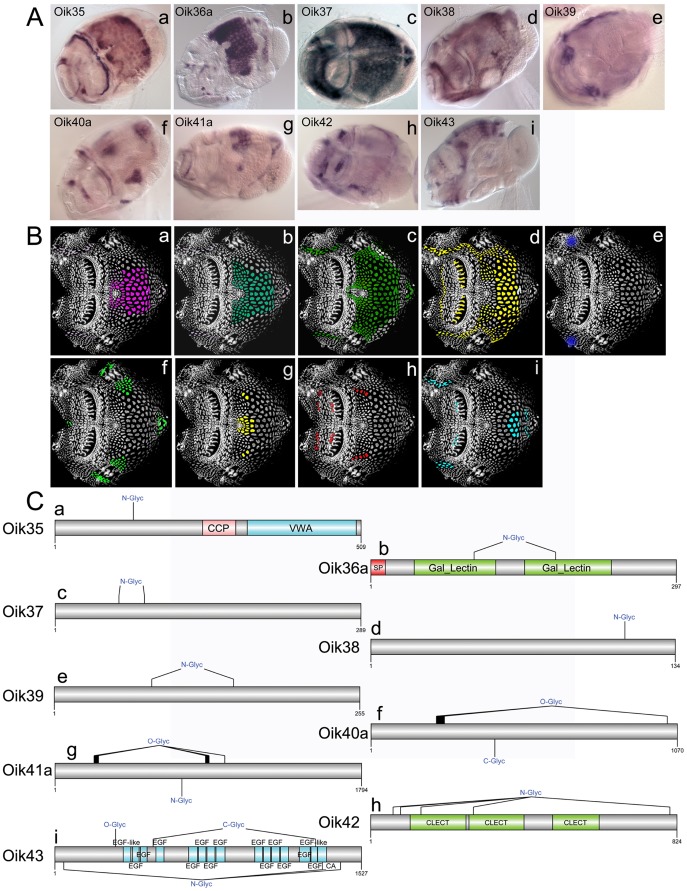
Oikosins expressed in diverse regions of the epithelium. (A) *In situ* hybridisation patterns for oikosins: a) oik35, b) oik36a, c) oik37, d) oik38, e) oik39, f) oik40, g) oik41a, h) oik42, and i) oik43. B) Oikosin expression patterns in A are indicated by corresponding coloured domains on epithelial spreads (anterior to left). C) Protein domain and modification schemas are shown for oikosins 35–43: Sp, signal peptide; C-, N- or O-Glyc, predicted C, N and O glycosylation sites; CCP, complement control protein modules, also known as short consensus repeats SCRs or SUSHI repeats; VWA, von Willenbrand factor type A domain. Gal_Lectin, galactose binding lectin domain; CLECT, c-lectin domain; EGF, epidermal growth factor domain; EGF_CA, calcium binding epidermal growth factor-like domain. *In situ* images are oriented with the oral cavity towards the left and were performed on day 3 animals with trunk lengths ranging from 350–400 µm in size.

**Figure 6:**
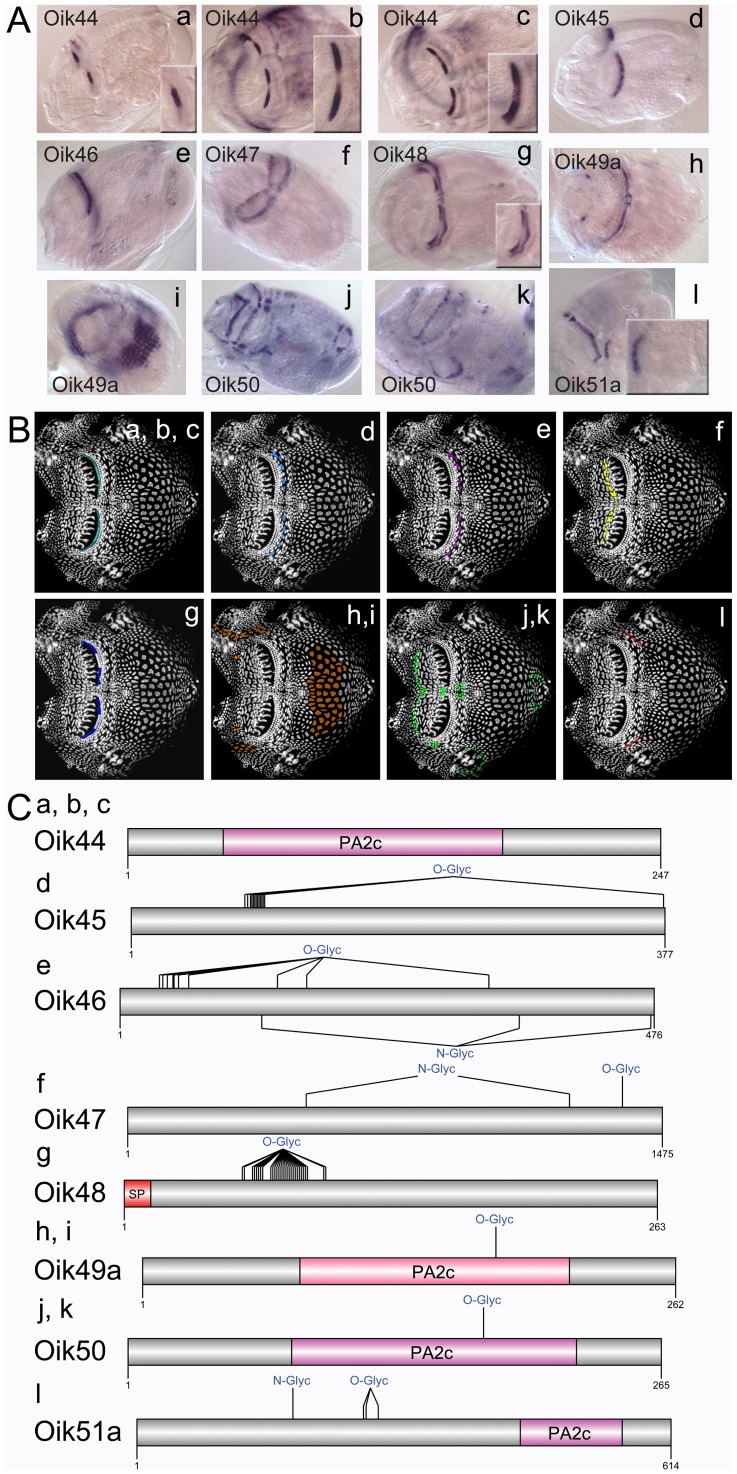
Oikosins expressed in bands or spots. (A) *In situ* hybridisation patterns for oikosins: a,b,c) oik44, d) oik45, e) oik46, f) oik47, g) oik48, h,i) oik49a, j,k) oik50, and l) oik51a. B) Oikosin expression patterns in A are indicated by corresponding coloured domains on epithelial spreads (anterior to left). C) Protein domain and modification schemas are shown for oikosins 44–51: Sp, signal peptide; N- or O-Glyc, predicted N and O glycosylation sites; PA2c, phospholipase A2 domain. *In situ* images are oriented with the oral cavity towards the left and were performed on day 3 animals with trunk lengths ranging from 350–400 µm in size.

Oikosins 35–43 were expressed in a diverse array of patterns in the epithelium (**Fig. 5**) The von Willebrand factor type A (VWA) domain found in oik35, is present in plasma proteins including complement factors, integrins and collagen subtypes. It can bind various ligands, participating in numerous biological processes such as cell adhesion, migration, homing, pattern formation, and signal transduction [25], [26]. Oikosins 44 to 51 exhibited more restricted expression domains in spots and bands (**Fig. 6**). Oikosins 45 and 46 were expressed in rows of posterior Fol cells, adjacent to the posterior of the 3 rows of Nasse cells, and oik47 was produced in a line of cells delineating the anterior and giant Fol cells. Oikosins, 44 and 48 exhibited intriguing gradient expression patterns distributed laterally along the anterior row of Nasse cells. Four oikosins, (44, 49–51), contain phospholipase A2 domains that are widespread in both animal and plant kingdoms. Of these, oik44 *in situs* exhibited different, but related, patterns restricted to the Nasse cells within the same set of experiments: some specimens had signals in the central cells (**Fig. 6a**), others in the cells flanking either side of this central region (**Fig. 6b**) and a third group showed a merged pattern (**Fig. 6c**), likely representing the transition from one pattern to the other. Together with oik45, oik44 is predicted to be GPI-anchored, making them potential candidates as proteins that could link cell membrane or sub-membrane mediated processes aiding in scaffolding of house construction (see Fig. 9 in [8]). Oik49, exhibited alternative expression patterns with some animals staining in two bilaterally symmetric spots anterior to the Fol region (**Fig. 6h**) whereas others appeared to be in a different phase of house synthesis, exhibiting a saddle-like expression pattern (**Fig. 6i**). Oik50 also had alternative patterns, with expression at the anterior end of the anterior Fol cells, in cells between the two sets of giant Fol cells, and at the anterior end of the anterior rosette (**Fig. 6j**), or alternatively, in a row of cells abutting the lateral and ventral borders of the giant Eisen cell region (**Fig. 6k**). Oik51 was expressed in a single row of cells located between the field of Martini and the Chain of Pearl cells on the lateral sides of the eptihelium (**Fig. 6l**).

Oikosins 44, 48 and 50 were the only oikosins to show alternate expression patterns in the epithelium. During the house building phase of the life cycle, all other oikosin mRNAs were observed in their respective oikoplastic epithelial domains in all specimens examined, suggesting that on-off expression of oikosins is not the mechanism through which definition of individual pre-house rudiments is determined. It is currently unclear whether the alternate expression patterns of oik44, 48 and 50 are implicated in defining initial, intermediate and end points of individual pre-house rudiment construction or whether this alternance serves some other role in the formation of the intricate 3D structure of the house.

### The *Oikopleura* House: on the Front lines of Innate Immune Defence?

Secreted oikosins 44, 49, 50 and 51 contain phospholipase A_2_ domains. Secretory phospholipase A_2_s (sPLA_2_) hydrolyze the sn-2 position of glycerophospolipids, triggering signaling events to regulate cell functions, inflammation and destruction of bacteria as part of an innate immune defence [27]. They also have roles in generating reactive oxygen species, cytotoxicity, cell migration, apoptosis, proliferation, differentiation and cancer [27]. All of these oikosins are expressed in cell fields associated with the Fol region, responsible for production of the *fcf*, as are oik21a,b, proteins which contain a perxoxidase domain linked to immunity mechanisms in other invertebrates (see above). Oik49 also exhibits other expression domains, contributing more to the shell of the house. In humans, sPLA_2_s are an important part of the innate immune system in the digestive tract, acting to regulate the intestinal microflora [28]. In *Oikopleura*, the *fcf* is intimately associated with the digestive process, as it is here that food particles are initially collected on the filters and brought to the mouth. Thus, it is intriguing that a series of proteins with domains implicated in innate immunity are secreted from a region of the epithelium that produces this structure, and could indicate that they are an early defensive measure against the variety of marine bacteria the animal encounters in its pelagic environment. As *O. dioica* is known to be able to feed on bacteria [29], and has a rapid gut passage time of several minutes, an alternative explanation could be that the oikosins containing PLA_2_ domains serve a role in the pre-digestion of these particles through the disruption of bacterial cell walls.

### Genomic Organization and Phylogeny of Oikosins

**Figure 7:**
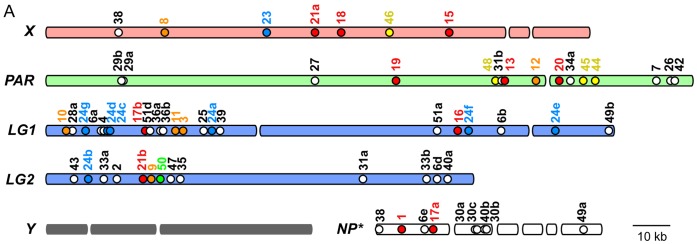
Location of oikosins on *Oikopleura dioica* chromosome scaffolds (based on Denoued et al., 2010). X and Y sex-specific regions are linked to a large pseudo-autosomal region (PAR) through physical junctions that are not yet identified. Autosomal linkage groups (LG1 and LG2) are also shown. NP*: assemblies of scaffolds that can not yet be placed on the above maps. Breaks in the X, Y, PAR and LG1 and LG2 maps indicate areas where physical links between contigs of scaffolds were of lower confidence. Oikosin gene locations are colour-coded according to their expression regions in the oikoplastic epithelium (see Fig. 1). Those indicated as white circles do not fall into any of the specific defined cell fields of Fig. 1 and likely contribute to formation of the overall shell of the house. Oikosin loci that are not indicated, fall on scaffolds <25 kb, which were not mapped.

Based on the draft genome assembly [30] we were able to map 57 of the 80 oikosin gene loci to chromosomal scaffolds (**Fig. 7**) and an additional 9 oikosins to scaffold sub-assemblies that have not yet been placed among the linkage groups. No oikosin loci map to the Y chromosome, suggesting that there are no sex-specific modifications of the house. This concurs with a lack of any perceived morphological differences between the houses of male and female *O. dioica* (note also that all other appendicularians are hermaphroditic). With the exception of loci (*oik44–48*) expressed in the Nasse cells, which are restricted to the X and pseudoautosomal regions, oikosin loci expressed from all other major cellular fields in the oikoplastic epithelium are distributed across the non-Y chromosomal scaffolds. This organization indicates a lack of any *cis*-regulatory enhancers or locus control regions implicated in coordinating expression from a given cellular field, even in those with very restricted cell numbers such as the 7 cells of the giant Fol (*oik13–21b*) or the 4 giant lateral cells of the Eisen (*oik23–24g*). One terminal segment of autosomal LG1, however, is particularly rich in paralogs of diverse oikosin families, including those that remain in close association, such as *oik24c,d* and *36a,b*, the former being the progeny of the most recent duplication event in an 8-paralog family (**Fig. S5**). Thus, this chromosomal region may be particularly rich in the birth of new oikosins through gene duplication events, though such events do not appear to be restricted to this region (see *oik29a,b* on the pseudoautosomal region). On the other hand, oikosin loci are not found to be closely spaced on the X chromosome, and only one paralog is observed (*oik21a*), suggesting that oikosin gene duplication events are much less frequent in this region of the genome. Nonetheless, genes for each of the main sub-oikoplastic fields are found on this chromosome.

**Figure 8:**
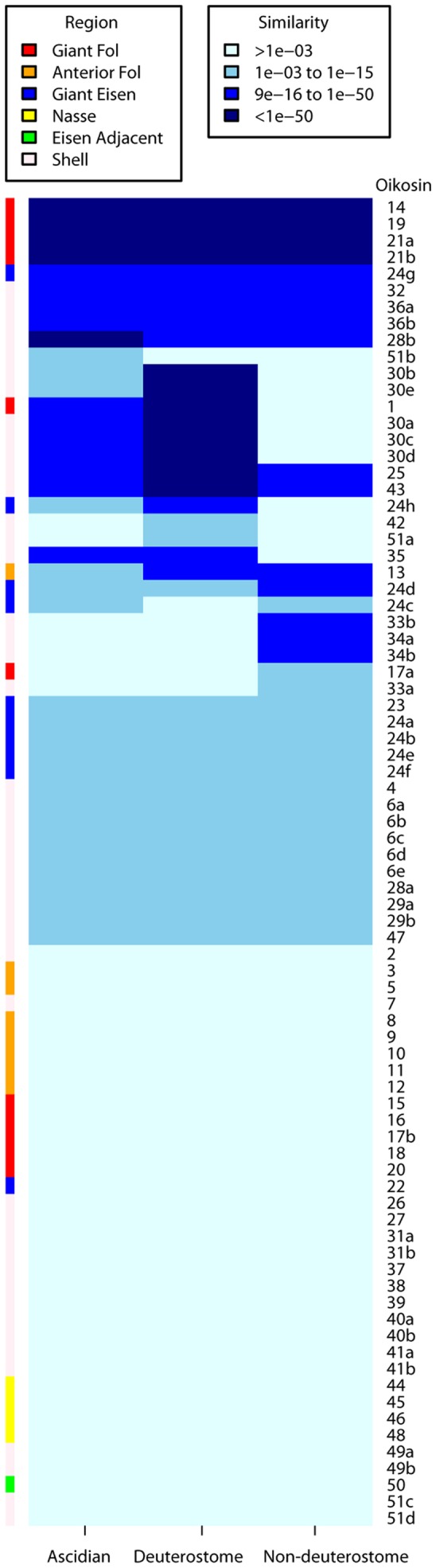
Similarity of oikosins to known proteins in ascidians, deuterostomes and non-deuterostomes. The location of expression of the corresponding oikosin genes is denoted at the left according to the “Region” legend above with color coding as in Fig. 1. Degree of similarity was binned in 4 categories as defined in the “Similarity” legend above based on e-values. More details concerning the homologies are given in **Tables S4** and **S5**.

All three of the urochordate sister classes, the appendicularians, the ascidians and the thaliaceans, share a common strategy of erecting extracellular structures based on a cellulose scaffold [5]. Morphologically, however, these structures are quite divergent. This appears also to be reflected in their respective molecular compositions (**Fig. 8** and **Tables S4** and **S5**). Of the 80 oikosins identified to date, only two, oik28b and oik51b, exhibit higher similarity to ascidian proteins than they do to other deuterostome or non-deuterostome proteins. Fully 35 oikosins (44%) show no similarity to any known proteins, suggesting their *de novo* appearance in the appendicularian lineage, or alternatively, that they have diverged to such an extent that their ancestry is no longer detectable. Among oikosins produced from epithelial regions topologically associated with the *fcf*, there is an interesting dichotomy. Those produced from the giant Fol cells are almost equally divided between the only group (oik14, 19, 21a,b) that shows the highest degree of similarity to proteins across the three phylogenetic categories and those showing no similarities (oik15, 16, 17b, 18 and 20) to known proteins. In contrast, all 4 of the oikosins produced from the Nasse cells are novel proteins as are 7 of the 8 oikosins expressed from the anterior Fol, the exception being oik13. Oikosins produced from the Eisen region, responsible for production of the *if*, do not show an affinity for any particular degree of evolutionary relationship, and this is also characteristic of oikosins produced from regions of the house that are not topologically associated with either of the filter sets. Since proteins produced from the giant Fol cells, implicated in production of the *fcf* show the deepest evolutionary roots, it may be that following acquisition of cellulose synthesis capability at the base of the tunicate lineage [5], a basic elaboration of the *fcf* may have been one of the earliest steps in the evolution of the current house structure in the appendicularian lineage.

### Oikosins Produced in Different Cellular Fields can Colocalize in the Extracellular Pre-house Matrix

**Figure 9:**
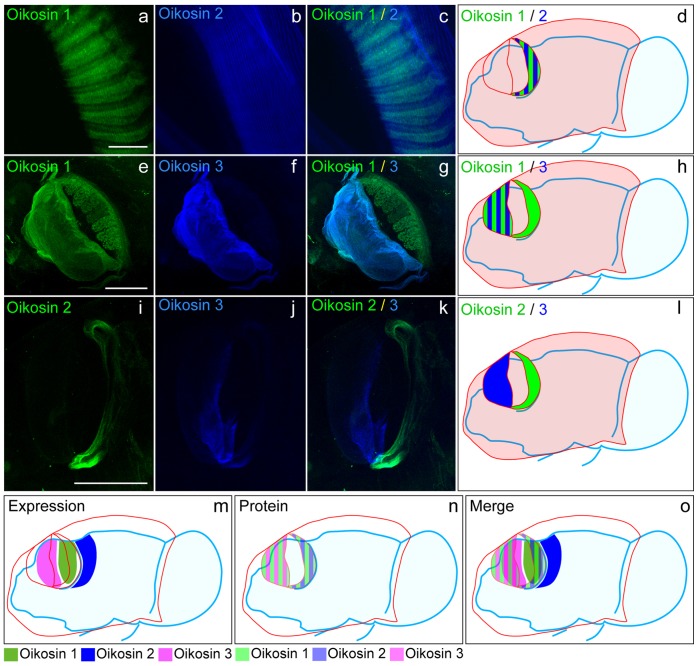
Localisation of oikosins 1, 2 and 3 in the *fcf* (food-concentrating filter) in pre-house rudiments. Panels in the top row (a-c) display co-staining of oikosins 1 and 2 in the inflating *fcf,* (anterior to upper left). Scale bar: 10 µm. Panels in the middle rows (e-g and i-k) show co-staining of oikosins 1 and 3, and oikosins 2 and 3, respectively (anterior to left), in the nascent secreted pre-house. Scale bars: 100 µm. d, h, and l) Cartoons illustrating the location of the stained structures in the pre-house rudiment (pink) superposed on an outline of a lateral view of the animal (blue), anterior to the left. Striped regions indicate regions of antibody co-staining. In the bottom panels the composite mRNA expression domains (m, dark shades) and protein localization in the rudiment (n, translucent pastel shades) of oikosins 1,2 and 3 are shown as well as a merged view of both (o).

Oik1 is expressed exclusively in the giant Fol cells. Oik2 has a more complex pattern including several rows of peri-oral cells in the anterior epithelium, a single row of cells surrounding the anterior Fol region, three rows of Nasse cells, the posterior Fol, the chain of pearls, and the 3 central cells in the giant Eisen field. Oik3 is expressed only from the anterior Fol cells [10]. Antibodies raised against these three oikosins reveal that they all participate structurally in the formation of the *fcf*, produced above the Fol region (**Fig. 9**). Oikosins 1 and 3, produced from adjacent cellular fields show extensive colocalisation in nascent and mature pre-house rudiments and are involved in forming the *fcf* particle sieving meshwork. Oik2, shows a spatially distinct pattern in nascent rudiments but comes to surround the oik1/3 colocalised structure with a fine array of long fibrils in the mature rudiment, helping to define the outer boundary of the *fcf* basket. Thus, oikosins can migrate in the extracellular matrix, over regions of at least up to 20 cell diameters. Their extracellular localisations are guided in part by association with the cellulose microfibrillar scaffold [8] and by oikosin-oikosin interactions. Domains involved in directing the specificity of the latter type of interactions remain to be identified.

### Heparan Sulfate Proteoglycans are Required for Inflation of Pre-house Rudiments

Heparan sulfate (HS), a strongly anionic linear polysaccharide, is a constituent of proteoglycans, where 2–3 HS chains are covalently attached to extracellular matrix and cell surface proteins [31]. HS is present in all animal tissues with deep evolutionary roots extending to the cnidarians, though it is absent in poriferans. HS binds to a variety of protein ligands and regulates numerous biological processes in development, angiogenesis, tumour metastasis, coagulation and defence. Proteoglycans have net negative charge that attracts positively charged Na^+^ ions, in turn attracting water molecules, to maintain the ECM and associated cells in a hydrated state. Given this property, we examined whether proteoglycans might play a role in regulating hydration and inflation of *Oikopleura* pre-house rudiments.

**Figure 10:**
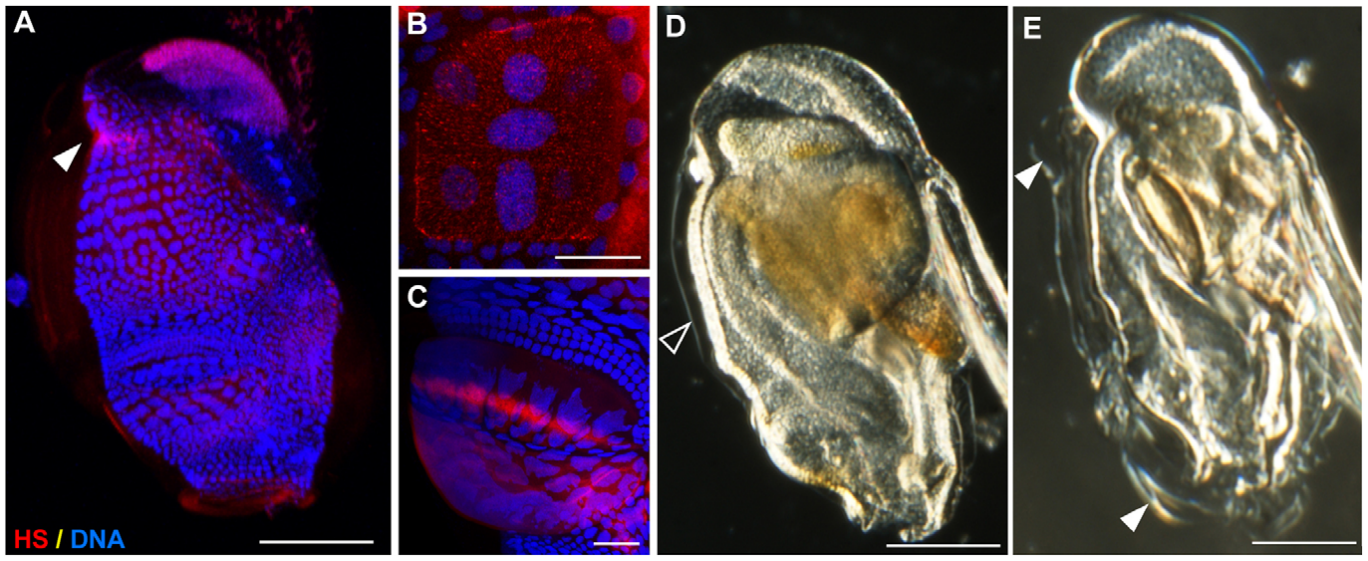
Arrest of pre-house rudiment inflation in the presence of sodium chlorate. (A,B,C) Localization of heparin/heparan sulfate (HS) on pre-house rudiments. Though generally present throughout the rudiment, more intense staining was detected in a posterior dorsal region (A, filled arrowhead), a region in which pre-house inflation is normally initiated, and corresponding spatially to the upper blister observed during failed inflation in E. HS staining was also enhanced along mesh lines of the nascent *if* (inlet filter) produced above the Eisen region (B). HS staining was also enhanced above the anterior and giant Fol regions (C) responsible for production of the *fcf* (food-concentrating filter), and corresponding spatially to the lower blister observed during failed inflation in E. (D,E) Day-5 animals were cultured at room temperature in seawater in the absence (D) and presence (E) of 3 mM sodium chlorate, an inhibitor of glycosaminoglycan sulfation, for 9 h. The animals are oriented anterior (mouth) to bottom, posterior (gonad) to top, and ventral (tail) to right. Pre-house rudiments stacked on the trunk of the animals are indicated with arrowheads. When glycosaminoglycan sulfation was reduced, only partial inflation of house rudiments (open arrowhead, (D) was observed in localized blisters (filled arrowheads, E). Scale bars: 200 µm for A, D and E, and 50 µm for B and C.

All glycosaminoglycans are O-sulfated and HS are in addition, N-sulfated on glucosamine residues. Enzymes that add these sulfate residues use 3′-phosphoadenosine5′-phosphosulfate (PAPS) as the sulfate donor. If PAPS synthesis is impaired by sodium chlorate, a competitive inhibitor, the sulfation reactions will not proceed. We first examined HS in the house pre-rudiments (**Fig. 10**) using an anti-heparin/heparan sulfate monoclonal antibody. HS was distributed throughout the pre-house rudiment but was notably intensified in a dorsal region, where inflation of pre-house rudiments commences (**Fig. 10a**), in the meshes of the *if* above the giant Eisen cells (**Fig. 10b**) and above the anterior and giant Fol regions (**Fig. 10c**) responsible for production of the *fcf*. Interestingly, the Oik24 family (8 paralogs), secreted by the 4 lateral giant Eisen cells, all contain a Zn-dependent metalloprotease domain, known to mediate shedding of proteoglycan ectodomains [31]. It is at present unclear if such shedding might occur during expansion of the *if* meshes as houses are inflated or if this may play some other role at the location where seawater first enters the *Oikopleura* house. We then exposed lots of day 5 animals (n = 13 in each lot) to 0.3, 3, 10 and 30 mM sodium chlorate in seawater. No effects were observed at 0.3 mM and a 30 mM dose was uniformly lethal. At 10 mM, 54% of the animals died and the remaining 46% were unable to inflate their houses. At 3 mM, lethality was reduced to 15% and the remaining 85% were still unable to inflate their prehouses. As in untreated control animals (**Fig. 10d**), pre-house rudiments did form around the trunk of the exposed animals but inflation was arrested, with only small blisters observed above the dorsal posterior initiation region and above the anterior Fol (**Fig. 10e**). When animals (2 lots of 20) were incubated in seawater with the addition of 3 mM NaCl, to mimic the increase in salt concentration of 3 mM sodium chlorate, no effects on mortality or house inflation were observed as would be expected for the euryhaline *O. dioica* in response to this very small salt increase (0.5%). Therefore, inhibition of proteoglycan sulfation arrests normal hydration and expansion of compact *Oikopleura* pre-house rudiments.

### Conclusions

Based on 2D electophoretic separation of proteins isolated from pre-house rudiments [10] we have now identified a majority of the proteins constituting the *Oikopleura* house. The common urochordate strategy of elaborating extracellular structures founded on a cellulose scaffold diverges significantly in the morphological design of the house/tunic among the three urochordate classes. Two of these classes remain pelagic throughout the life cycle whereas one (ascidians) is principally a sedentary benthic group with only a short pelagic larval phase. The morphological design differences are now also evident at the molecular level: appendicularian, *O. dioica* oikosins have very little in common with the ascidian, *Ciona intestinalis* proteome. It would be of interest as genome data becomes available on thaliaceans, to determine if any higher level of similarity is observed in the two wholly pelagic classes. Almost half of the oikosin complement exhibit no known domain structures or other similarities to known proteins, suggesting *de novo* appearance in the appendicularian lineage. Known domains are implicated in cellulose binding, protein-protein interactions or sPLA_2_ activity. Production of the latter is concentrated in epithelial regions associated with construction of the *fcf*, suggesting a possible role of this structure in innate immune defence.

Gene duplication has been an important process in generating the current complement of house proteins with paralogs subsequently dispersed throughout the genome, though thus far, no sex-specific oikosins have been identified. This genomic organization seems incompatible with any common cis-regulatory enhancers or locus control of the coordinate regulation of oikosin gene expression, even from very specific and limited cell fields such as the 4 lateral giant cells of the Eisen region. Furthermore, the 3D spatial distribution of multiple loci of several oikosins in the extensively polyploid oikoplastic nuclei, appear to rule out possible *trans*-regulatory organizations such as polytene or other clustered configurations [32].

House architecture is defined in part by the production of specific oikosins in distinct spatial cellular fields within the oikoplastic epithelium but also involves migration of some oikosins in the extracellular space mediated by protein-protein and protein-cellulose interactions. With on the order of 100 proteins, the *Oikopleura* house offers a tractable model system to investigate how proteins of diverse degrees of evolutionary history have combined and diversified to create a complex, highly functional extracellular structure essential to filter-feeding, a strategy common to the entire urochordate lineage.

## Materials and Methods

### Animal Culture and Prehouse Rudiment Collection

*Oikopleura dioica* were cultured at 15°C [6]. Prior to house collection, animals were forced out of their houses and transferred into a 12 cm plastic dish with filtered seawater. After rinsing the animals several times with filtered seawater to remove residual food particles, swelling pre-house rudiments were collected from animals during the inflation process with a hooked needle. Collected rudiments were stored on ice, centrifuged to remove residual seawater and stored at −80°C.

### Nano-LC-LTQ-Orbitrap-MS

Proteins were extracted from lots of 100 rudiments [10], precipitated using a 2D Clean-Up kit (GE Healthcare) and resuspended in 2× Laemmli loading buffer (Sigma). Samples were run on 4% stacking/6% separating SDS-PAGE gels at 25 mA for a few minutes and Coomassie-stained. Two gel portions were excised, corresponding to fractions >75 kDa and <75 kDa. The two gel samples were cut into small pieces, digested with 0.1 µg trypsin (Promega, Madison, WI, USA) in 20 µl of 25 mM ammonium bicarbonate, pH 7.8 at 37°C for 16 h and purified using µ-ZipTips C18 (Millipore, Billerica, MA, USA). LC-MS was performed at the Proteomics Core Facility of the Biotechnology Centre, University of Oslo. The separation of peptides was performed using a Dionex Ultimate 3000 nano-LC (Sunnyvale CA, USA) connected to a linear quadrupole ion trap-Orbitrap (LTQ-Orbitrap) mass spectrometer (ThermoElectron, Bremen, Germany) equipped with a nano-ESI source. Liquid chromatographic separation was achieved on an Acclaim PepMap 100 column (C18, 3 µm, 100 Å) (Dionex, Sunnyvale CA, USA) capillary of 12 cm bed length. Solvent gradient: 7% B to 35% B in 77 minutes and 35% B to 50% in 10 minutes with a flow rate of 300 nL/min. Solvent A: 0.1% formic acid. Solvent B: aqueous 90% acetonitrile in 0.1% formic acid. The mass spectrometer was operated in the data-dependent mode to automatically switch between Orbitrap MS and LTQ-MS/MS acquisition. This allowed sequential isolation of up to six of the most signal intense ions for fragmentation on the linear ion trap using collision-induced dissociation (CID). For accurate mass measurements the lock mass option was enabled in MS mode [33]. Target ions already selected for MS/MS were dynamically excluded for 60 seconds. Further details about instrument parameters were previously described [34].

### Data Analyses

Raw data were processed using Thermo Proteome Discoverer software (v. 1.0 build 43) to generate Mascot generic files (*.mgf). A search against *Oikopleura* proteins (18199 sequences) was performed using MS/MS ion search algorithms from the Mascot house server (v2.2.1) by database comparisons [35]. Mass tolerances of 10 ppm for the precursor and 0.5 Da for MS/MS fragments were applied. The enzyme parameter in the MS/MS ions search form was set to trypsin (allowing one missed cleavage site), the peptide charge setting was 2+ and 3+, and variable protein modification parameters allowed methionine oxidation and N-terminal protein acetylation. The significance threshold was set to p<0.05.

### Sequence Analyses

After Mascot searches, gene models were verified using ESTs (https://www.genoscope.cns.fr/secure-nda/Oikopleura). Predictions were edited with GENSCAN (http://genome.dkfz-heidelberg.de/cgi-bin/GENSCAN/genscan.cgi), Softberry (http://linux1.softberry.com/berry.phtml?topic=fgenesh&group=programs&subgroup=gfind) and assessed with the following prediction tools: signal peptides (SignalP 4.0, http://www.cbs.dtu.dk/services/SignalP/; [36]), non-classical secretion (SecretomeP 2.0; http://www.cbs.dtu.dk/services/SecretomeP/; [37]), subcellular localization (WoLF PSORT; http://wolfpsort.org/), presence of protein domains (Pfam; http://pfam.sanger.ac.uk/; SMART; http://smart.embl-heidelberg.de), glycosylation (NetOGlyc 2.0; [38]; NetCGlyc 1.0; [39]; http://www.cbs.dtu.dk/services/), and transmembrane prediction (Phobius; http://phobius.sbc.su.se/). Protein homology searches were performed at NCBI (http://www.ncbi.nlm.nih.gov/) using non-redundant databases. Oikosin protein schemata were produced with DOG 1.0.5 (http://dog.biocuckoo.org/) [40]. Accession numbers for all oikosins are given in **Data S1**.

We identified oikosin families using an all-against-all psi-BLAST search followed by clustering the oikosins using the resulting E-values corresponding to the top hit for each protein pair. Clusters of oikosins consisting of more than two sequences with E-values less than 1×10^−50^ were then extracted giving seven families containing a total of 35 proteins. A further 12 proteins formed related pairs. The remaining 33 proteins showed weak or no evidence of relatedness with other oikosins. We carried out multiple sequence alignments of protein and cDNA sequences of oikosins within each family using four programs: PRANK [41]; MUSCLE [42]; T-coffee [43] and ClustalW [44]. Poorly aligned regions were removed using Gblocks [45] before building phylogenetic trees with PhyML [46]. Phylogeny.fr [47] was used in part to organize the phylogenetic analysis workflow.

### Whole Mount *in situ* Hybridization

Specific probes with lengths between 250 and 800 bp were PCR amplified from an *O. dioica* cDNA library (day 2) (**Table S3**). PCR products were cloned into pCRII-TOPO (Invitrogen) and positive colony-PCR clones were sequenced. Antisense RNA probes were *in vitro* transcribed from 250 ng of sequence-verified colony PCR products with either T7 or SP6 RNA polymerase (BioLabs) in the presence of digoxigenin-labeled UTP (DIG RNA Labeling Mix; Roche) and precipitated. Day 3 animals were fixed in 4% paraformaldehyde, 0.1 M MOPS pH 7.5, 0.5 M NaCl and 0.1% TritonX at 4°C overnight (O/N). The specimens were rinsed with 0.1 M MOPS pH 7.5 and 0.5 M NaCl and then stored in 70% ethanol at −20°C. Prior to hybridisation, animals were rehydrated in 0.1 M MOPS pH 7.5, 0.5 M NaCl, washed in 50 mM Tris-HCl pH 8, treated 5 min with 10 µg/ml proteinase K at 37°C and incubated in 0.1 M MOPS pH 7.5, 0.5 M NaCl for 20 min at room temperature (RT). Hybridizations and detection of probes were performed as described [48].

### Immunolocalisation

Rabbit antisera against oikosins 1, 2 and 3 were generated by Washington Biotechnology Inc. (Baltimore, MD). To localize oikosins in pre-house rudiments, day 4 animals were fixed in 4% paraformaldehyde/0.1 M MOPS pH 7.5/0.5 M NaCl at 4°C O/N. Fixed animals were rinsed with PBS/0.1% Tween 20 (PBS-T), and then blocked with 3% BSA+PBS-T at 4°C O/N. Samples were incubated at 4°C O/N with antisera (1∶100 dilution) against oikosins 1–3 in 3% BSA+PBS-T, washed with PBS-T, post-fixed in 4% paraformaldehyde/0.1 M MOPS pH 7.5/0.5 M NaCl for 30 min at RT, washed as after the first fixation and incubated at 4°C O/N with anti-rabbit IgG conjugated Alexa 568 (1∶1000) in 3% BSA+PBS-T. Samples were washed with PBS-T and then mounted in Vectashield to be analyzed with a Leica TCS laser scanning confocal microscope using Leica (LAS AF v2.3) software.

### Heparin/heparan Sulfate Staining and Reduced Sulfation of Glycosaminoglycan Synthesis

Day 5 animals were fixed in 4% paraformaldehyde/0.1 M MOPS pH 7.5/0.5 M NaCl at 4°C O/N. Fixed animals were rinsed with PBS/0.1% Tween 20 (PBS-T), and then blocked with 3% BSA+PBS-T at 4°C O/N. Heparin/heparan sulfate content was probed by incubating in 1% BSA+PBS-T containing mouse anti-heparin/heparan sulfate monoclonal antibody (1∶100, MAB2040, Millipore) at 4°C O/N, followed by incubation in Rhodamine Red X conjugated goat anti-mouse IgG (1∶200 in 1% BSA+PBS-T) at 4°C O/N. Nuclei were stained with 1 µM To-Pro-3 iodide (Molecular Probes). Specimens were mounted and analyzed by confocal microscopy as above. To reduce sulfation of glycosaminoglycans, a stock 1 M solution of sodium chlorate (Sigma) was prepared in seawater. Day 5 animals were cultured for 9 h in seawater containing crushed algae and 3 mM sodium chlorate or 3 mM NaCl. After incubation, animals were anaesthetized with 0.2 mg/ml MS-222 (Sigma) for photography.

## Supporting Information

Figure S1 **Oikosins expressed in anterior Fol cells.** Anterior Fol cells are indicated by orange labeling of their nuclei on an epithelial spread (dorsal view, oral side on the left). a-e: *in situ* hybridisation patterns of oikosins: a) oik8, b) oik9, c) oik11, d) oik12, e) oik13. Protein schemas of the respective oikosins are shown in Fig. 2. *In situ* images are oriented with the oral cavity towards the left and were performed on day 3 animals with trunk lengths ranging from 350–400 µm in size.
(PDF)

Figure S2 **Oikosins expressed in giant Fol cells.** The giant Fol cells are indicated by red labeling of their nuclei on an epithelial spread (dorsal view, oral side on the left). a-g: *in situ* hybridisation patterns of oikosins: a) oik14, b) oik15, c) oik16, d) oik18, e) oik19, f) oik20; g) oik21a. Protein schemas of the respective oikosins are shown in Fig. 2. *In situ* images are oriented with the oral cavity towards the left and were performed on day 3 animals with trunk lengths ranging from 350–400 µm in size.
(PDF)

Figure S3 **Oikosins expressed in the four lateral giant Eisen cells.** The four lateral giant Eisen cells are indicated by blue labeling of their nuclei on an epithelial spread (dorsal view, oral side on the left). a,b: in situ hybridisation patterns of oikosins: a) oik22, b) oik23. Protein schemas of the respective oikosins are shown in Fig. 3. *In situ* images are oriented with the oral cavity towards the left and were performed on day 3 animals with trunk lengths of 370 µm in size.
(PDF)

Figure S4 **Protein schemas for additional members of multigene oikosin families not shown in the core manuscript.** Sp, signal peptide; C-, N- or O-Glyc, predicted C, N and O glycosylation sites; An-peroxidase, peroxinectin_like animal heme peroxidase domain; ZnMc, Zinc-dependent metalloprotease domain; LDLa, Low-density lipoprotein receptor domain class A; EGF-like: epidermal growth factor-like domain; CUB, extracellular CUB domain; Tryp_SPc, Trypsin-like serine protease; ZP, zona pellucida domain; CCP, complement control protein modules, also known as short consensus repeats SCRs or SUSHI repeats; VWA, von Willenbrand factor type A domain. Gal_Lectin, galactose binding lectin domain; EGF, epidermal growth factor domain; EGF_CA, calcium binding epidermal growth factor-like domain. EGF-like, epidermal growth factor-like domain; VWD, von Willebrand factor type D domain; CLECT, c-lectin domain; ShKT, ShK toxin domain; ZnF_RBZ, Zinc finger domain in Ran-binding and other proteins; PA2c, phospholipase A2 domain.
(PDF)

Figure S5 **Relationships within oikosin families.** Families were identified as described in materials and methods. Alignments of cDNA and protein sequences were made using PRANK, MUSCLE, T-coffee and ClustalW, and phylogenetic trees were constructed using PhyML after using Gblocks to remove poorly aligned regions. Here, the results using PRANK are shown. Similar results were obtained using the other alignment programs. Where oikosins have the same numbers, all family members have the same expression domain, when they differ in expression domains they were accorded different numbers.
(PDF)

Table S1 SignalP 4.0 prediction of signal peptides in oikosins. Definitions of scores/positions can be found at http://www.cbs.dtu.dk/services/SignalP-4.0/output.php. A D-score above 0.450 predicts a signal peptide (**Y**) whereas a score below this threshold does not (n).
(PDF)

Table S2 SecretomeP 2.0 prediction of secretion of oikosins not predicted to have a signal peptide by SignalP 4.0. Non-classically secreted proteins should have an NN-score exceeding a threshold of 0.5.
(PDF)

Table S3 Primers generating *in situ* probes between 250 and 800 bp for newly identified oikosins.
(PDF)

Table S4 BLASTp similarities of oikosin proteins to *Ciona intestinalis* and deuterostomes. Oik, oikosin; % cov, % coverage; e-val, BLASTp e-value; Non-deut e-val, lowest e-value from **Table S5** for the given oikosin; -, no similarities found.
(PDF)

Table S5 BLASTp similarities of oikosin proteins to non-deuterostome organisms. Oik, oikosin; % cov, % coverage; e-val, BLASTp e-value; -, no similarities found.for the given oikosin; -, no similarities found.
(PDF)

Data S1 Accession numbers for oikosins.
(PDF)
