# Supplementary material for: The Evolving Proteome of a Complex Extracellular Matrix, the Oikopleura House
Source: PLoS One. 2012 Jul 5;7(7):e40172. doi: 10.1371/journal.pone.0040172 (PMC3390340; doi:10.1371/journal.pone.0040172)
Supplement: Figure S1 — Oikosins expressed in anterior Fol cells. Anterior Fol cells are indicated by orange labeling of their nuclei on an epithelial spread (dorsal view, oral side on the left). a-e: in situ hybridisation patterns of oikosins: a) oik8, b) oik9, c) oik11, d) oik12, e) oik13. Protein schemas of the respective oikosins are shown in Fig. 2. In situ images are oriented with the oral cavity towards the left and were performed on day 3 animals with trunk lengths ranging from 350–400 µm in size. (PDF) [file pone.0040172.s001.pdf]

SUPPORTING FIGURE S1

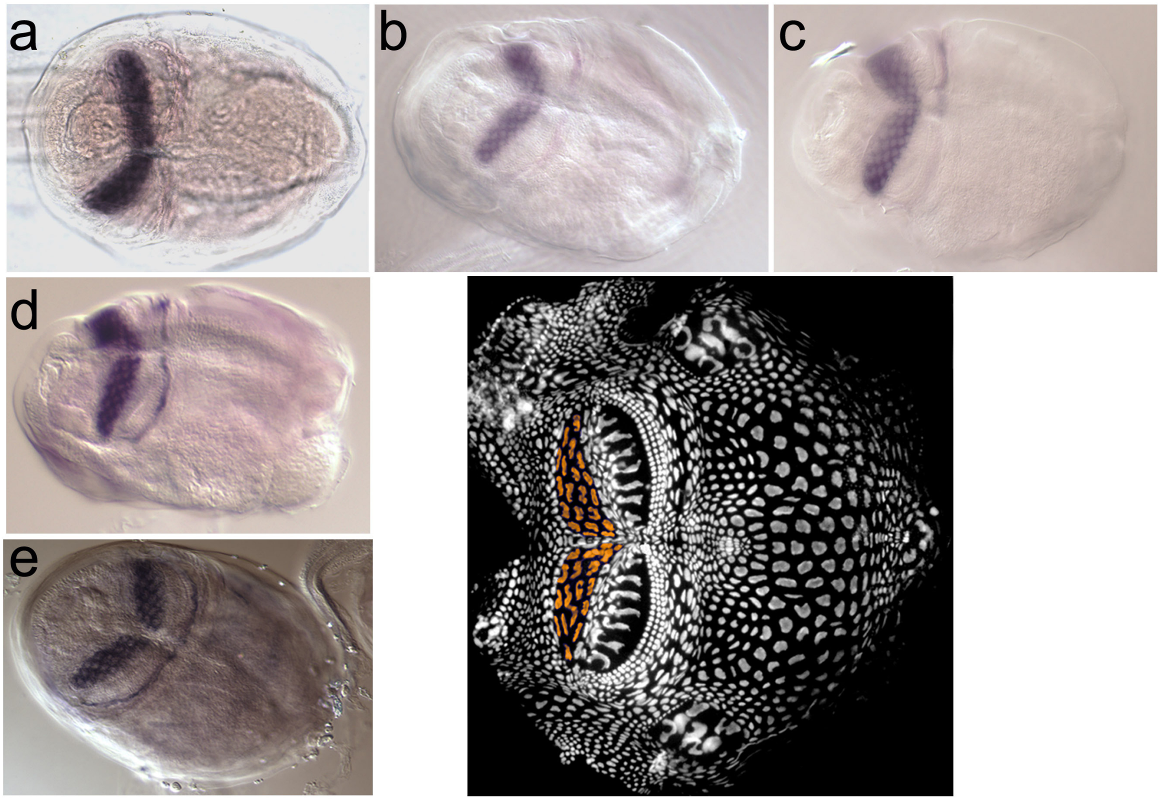

Figure S1. Oikosins expressed in anterior Fol cells. Anterior Fol cells are indicated by orange labeling of their nuclei on an epithelial spread (dorsal view, oral side on the left). a-e: *in situ* hybridisation patterns of oikosins: a) oik8, b) oik9, c) oik11, d) oik12, e) oik13. Protein schemas of the respective oikosins are shown in Fig. 2. *In situ* images are oriented with the oral cavity towards the left and were performed on day 3 animals with trunk lengths ranging from 350-400 μm in size.
