# Supplementary material for: The Evolving Proteome of a Complex Extracellular Matrix, the Oikopleura House
Source: PLoS One. 2012 Jul 5;7(7):e40172. doi: 10.1371/journal.pone.0040172 (PMC3390340; doi:10.1371/journal.pone.0040172)
Supplement: Figure S3 — Oikosins expressed in the four lateral giant Eisen cells. The four lateral giant Eisen cells are indicated by blue labeling of their nuclei on an epithelial spread (dorsal view, oral side on the left). a,b: in situ hybridisation patterns of oikosins: a) oik22, b) oik23. Protein schemas of the respective oikosins are shown in Fig. 3. In situ images are oriented with the oral cavity towards the left and were performed on day 3 animals with trunk lengths of 370 µm in size. (PDF) [file pone.0040172.s003.pdf]

SUPPORTING FIGURE S3

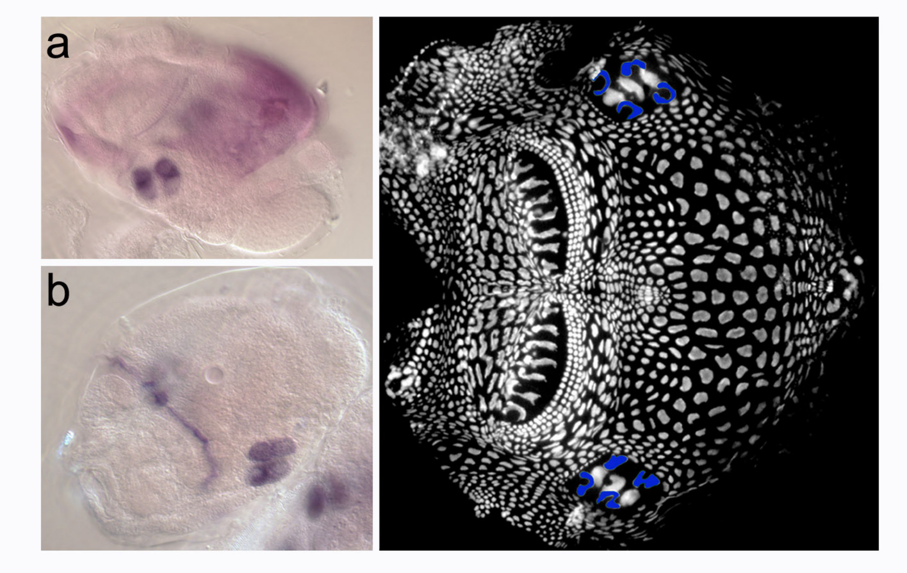

Figure S3. Oikosins expressed in the four lateral giant Eisen cells. The four lateral giant Eisen cells are indicated by blue labeling of their nuclei on an epithelial spread (dorsal view, oral side on the left). a,b: in situ hybridisation patterns of oikosins: a) oik22, b) oik23. Protein schemas of the respective oikosins are shown in Fig. 3. *In situ* images are oriented with the oral cavity towards the left and were performed on day 3 animals with trunk lengths of 370 μm in size.
