# Supplementary material for: The Evolving Proteome of a Complex Extracellular Matrix, the Oikopleura House
Source: PLoS One. 2012 Jul 5;7(7):e40172. doi: 10.1371/journal.pone.0040172 (PMC3390340; doi:10.1371/journal.pone.0040172)
Supplement: Figure S4 — Protein schemas for additional members of multigene oikosin families not shown in the core manuscript. Sp, signal peptide; C-, N- or O-Glyc, predicted C, N and O glycosylation sites; An-peroxidase, peroxinectin_like animal heme peroxidase domain; ZnMc, Zinc-dependent metalloprotease domain; LDLa, Low-density lipoprotein receptor domain class A; EGF-like: epidermal growth factor-like domain; CUB, extracellular CUB domain; Tryp_SPc, Trypsin-like serine protease; ZP, zona pellucida domain; CCP, complement control protein modules, also known as short consensus repeats SCRs or SUSHI repeats; VWA, von Willenbrand factor type A domain. Gal_Lectin, galactose binding lectin domain; EGF, epidermal growth factor domain; EGF_CA, calcium binding epidermal growth factor-like domain. EGF-like, epidermal growth factor-like domain; VWD, von Willebrand factor type D domain; CLECT, c-lectin domain; ShKT, ShK toxin domain; ZnF_RBZ, Zinc finger domain in Ran-binding and other proteins; PA2c, phospholipase A2 domain. (PDF) [file pone.0040172.s004.pdf]

Supporting Figure S4

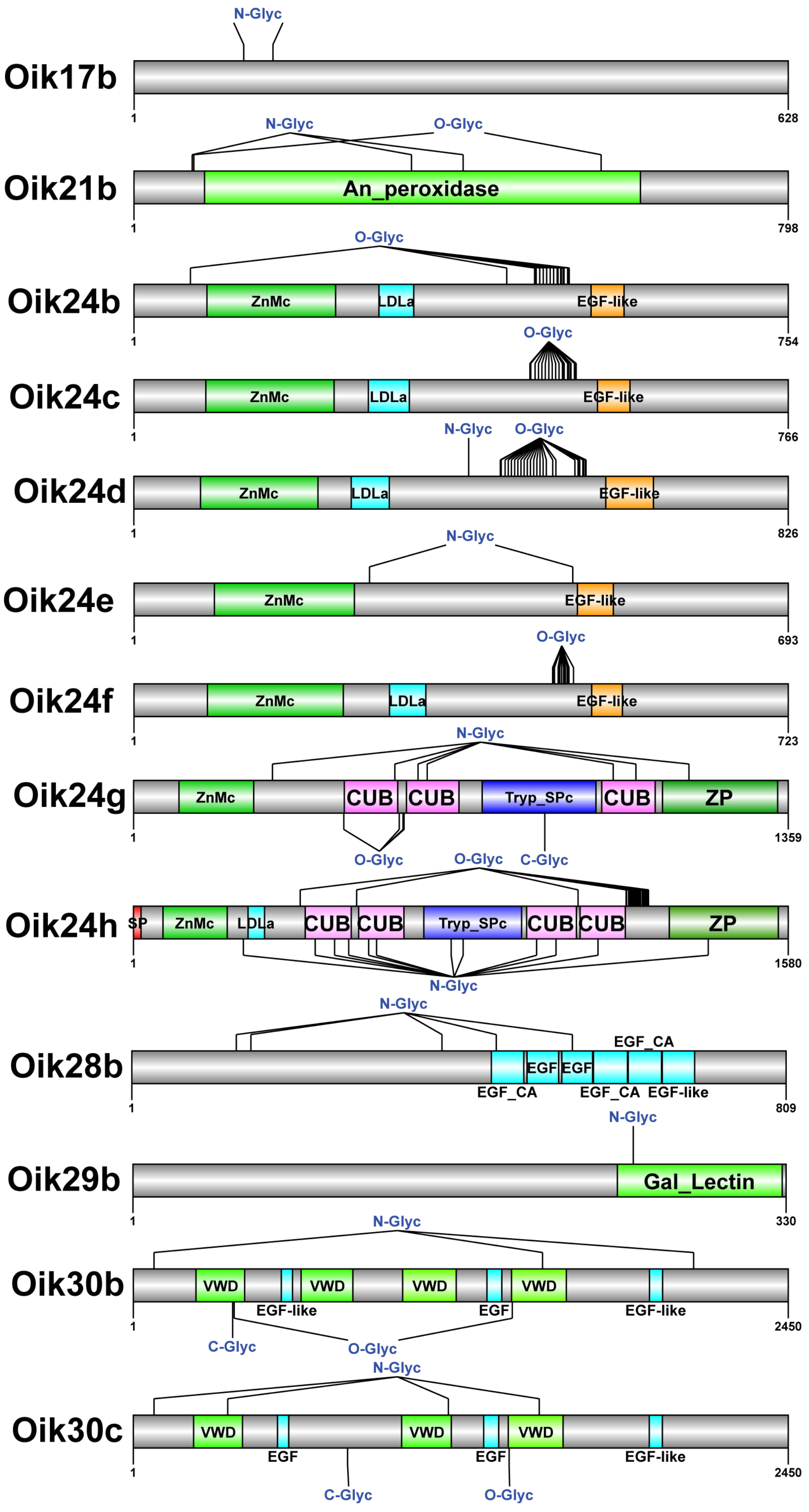

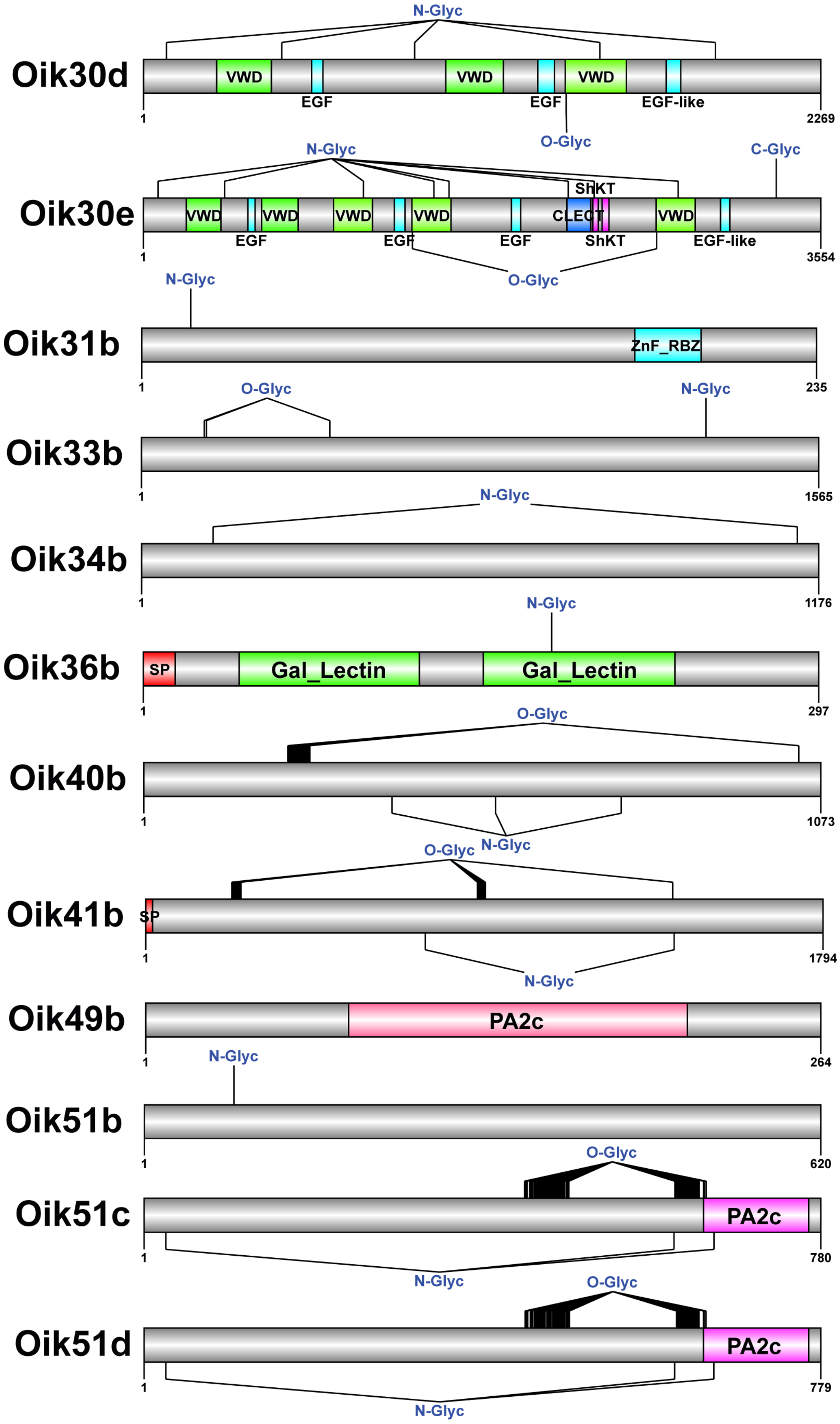

Oik30d
N-Glyc
VWD
EGF
VWD
EGF
VWD
EGF-like
O-Glyc
1
2269
Oik30e
N-Glyc
C-Glyc
ShKT
VWD
VWD
VWD
VWD
CLECT
VWD
EGF
EGF
EGF
ShKT
EGF-like
O-Glyc
1
3554
Oik31b
N-Glyc
ZnF_RBZ
1
235
Oik33b
O-Glyc
N-Glyc
1
1565
Oik34b
N-Glyc
1
1176
Oik36b
N-Glyc
SP
Gal_Lectin
Gal_Lectin
1
297
Oik40b
O-Glyc
N-Glyc
1
1073
Oik41b
O-Glyc
SP
N-Glyc
1
1794
Oik49b
PA2c
1
264
Oik51b
N-Glyc
1
620
Oik51c
O-Glyc
PA2c
N-Glyc
1
780
Oik51d
O-Glyc
PA2c
N-Glyc
1
779

Figure S4. Protein schemas for additional members of multigene oikosin families not shown in the core manuscript. Sp, signal peptide; C-, N- or O-Glyc, predicted C, N and O glycosylation sites; An-peroxidase, peroxinectin_like animal heme peroxidase domain; ZnMc, Zinc-dependent metalloprotease domain; LDLa, Low-density lipoprotein receptor domain class A; EGF-like: epidermal growth factor-like domain; CUB, extracellular CUB domain; Tryp_SPc, Trypsin-like serine protease; ZP, zona pellucida domain; CCP, complement control protein modules, also known as short consensus repeats SCRs or SUSHI repeats; VWA, von Willenbrand factor type A domain. Gal_Lectin, galactose binding lectin domain; EGF, epidermal growth factor domain; EGF_CA, calcium binding epidermal growth factor-like domain. EGF-like, epidermal growth factor-like domain; VWD, von Willebrand factor type D domain; CLECT, c-lectin domain; ShKT, ShK toxin domain; ZnF_RBZ, Zinc finger domain in Ran-binding and other proteins; PA2c, phospholipase A2 domain.
