# Supplementary material for: The Evolving Proteome of a Complex Extracellular Matrix, the Oikopleura House
Source: PLoS One. 2012 Jul 5;7(7):e40172. doi: 10.1371/journal.pone.0040172 (PMC3390340; doi:10.1371/journal.pone.0040172)
Supplement: Figure S5 — Relationships within oikosin families. Families were identified as described in materials and methods. Alignments of cDNA and protein sequences were made using PRANK, MUSCLE, T-coffee and ClustalW, and phylogenetic trees were constructed using PhyML after using Gblocks to remove poorly aligned regions. Here, the results using PRANK are shown. Similar results were obtained using the other alignment programs. Where oikosins have the same numbers, all family members have the same expression domain, when they differ in expression domains they were accorded different numbers. (PDF) [file pone.0040172.s005.pdf]

SUPPORTING FIGURE S5

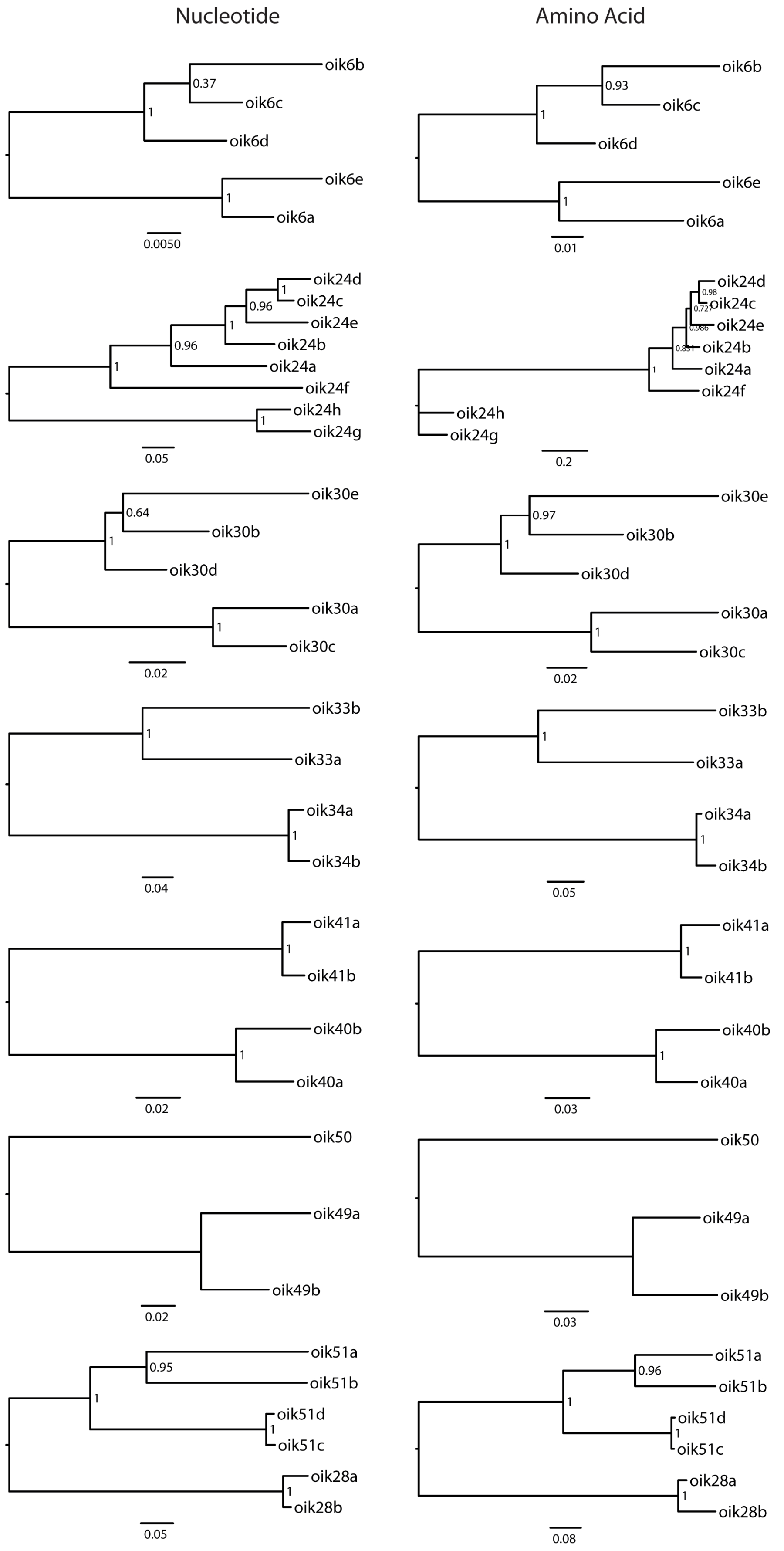

Figure S5. Relationships within oikosin families. Families were identified as described in materials and methods. Alignments of cDNA and protein sequences were made using PRANK, MUSCLE, T-coffee and ClustalW, and phylogenetic trees were constructed using PhyML after using Gblocks to remove poorly aligned regions. Here, the results using PRANK are shown. Similar results were obtained using the other alignment programs. Where oikosins have the same numbers, all family members have the same expression domain, when they differ in expression domains they were accorded different numbers.
