# Supplementary material for: The Evolving Proteome of a Complex Extracellular Matrix, the Oikopleura House
Source: PLoS One. 2012 Jul 5;7(7):e40172. doi: 10.1371/journal.pone.0040172 (PMC3390340; doi:10.1371/journal.pone.0040172)
Supplement: Table S1 — SignalP 4.0 prediction of signal peptides in oikosins. Definitions of scores/positions can be found at http://www.cbs.dtu.dk/services/SignalP-4.0/output.php. A D-score above 0.450 predicts a signal peptide (Y) whereas a score below this threshold does not (n). (PDF) [file pone.0040172.s006.pdf]

SUPPORTING TABLE S1

Table S1. SignalP 4.0 prediction of signal peptides in oikosins.

Oikosin	Cmax	Pos	Ymax	Pos	Smax	Pos	Smean	D	Signal Peptide
Oik1	0.336	15	0.557	15	0.949	6	0.918	0.752	Y
Oik2	0.123	17	0.295	17	0.828	6	0.682	0.504	Y
Oik3	0.425	22	0.357	22	0.467	6	0.300	0.326	n
Oik4	0.408	17	0.522	17	0.786	1	0.624	0.577	Y
Oik5	0.162	15	0.336	15	0.806	6	0.698	0.532	Y
Oik6a	0.109	30	0.148	5	0.255	3	0.235	0.195	n
Oik6b	0.109	41	0.149	5	0.261	3	0.240	0.199	n
Oik6c	0.108	16	0.156	5	0.279	3	0.261	0.213	n
Oik6d	0.112	19	0.162	4	0.287	3	0.273	0.222	n
Oik6e	0.109	25	0.147	5	0.252	3	0.232	0.193	n
Oik7	0.164	41	0.247	14	0.541	1	0.424	0.342	n
Oik8	0.143	19	0.208	19	0.454	6	0.302	0.259	n
Oik9	0.169	16	0.226	16	0.405	2	0.270	0.250	n
Oik10	0.128	14	0.277	14	0.761	6	0.654	0.481	Y
Oik11	0.150	20	0.291	15	0.727	6	0.614	0.465	Y
Oik12	0.141	31	0.219	14	0.448	1	0.326	0.277	n
Oik13	0.123	35	0.241	15	0.586	5	0.464	0.362	n
Oik14	0.516	17	0.579	17	0.761	13	0.648	0.616	Y
Oik15	0.109	54	0.106	2	0.113	35	0.109	0.108	n
Oik16	0.208	34	0.198	34	0.443	33	0.186	0.192	n
Oik17a	0.192	29	0.178	29	0.253	25	0.156	0.166	n
Oik17b	0.107	58	0.104	44	0.110	22	0.091	0.097	n
Oik18	0.189	18	0.377	18	0.842	7	0.742	0.574	Y
Oik19	0.240	16	0.439	16	0.888	6	0.789	0.628	Y
Oik20	0.123	21	0.261	8	0.637	1	0.582	0.434	n
Oik21a	0.134	14	0.232	14	0.527	4	0.398	0.321	n
Oik21b	0.114	18	0.153	5	0.227	1	0.214	0.186	n
Oik22	0.148	26	0.125	26	0.121	25	0.105	0.114	n
Oik23	0.114	16	0.121	35	0.162	23	0.127	0.124	n
Oik24a	0.236	17	0.390	17	0.750	6	0.617	0.513	Y
Oik24b	0.109	59	0.180	5	0.359	13	0.310	0.250	n
Oik24c	0.110	29	0.131	5	0.185	14	0.169	0.151	n
Oik24d	0.110	17	0.164	5	0.287	13	0.243	0.207	n
Oik24e	0.117	15	0.168	15	0.297	13	0.239	0.206	n
Oik24f	0.121	14	0.199	14	0.377	3	0.303	0.255	n
Oik24g	0.169	43	0.244	43	0.758	37	0.314	0.282	n
Oik24h	0.478	20	0.666	20	0.958	1	0.924	0.805	Y
Oik25	0.215	19	0.359	19	0.751	1	0.566	0.471	Y
Oik26	0.193	18	0.380	14	0.845	1	0.745	0.577	Y
Oik27	0.122	17	0.240	4	0.559	1	0.534	0.399	n
Oik28a	0.129	19	0.260	14	0.659	11	0.550	0.416	n
Oik28b	0.124	19	0.252	14	0.648	11	0.543	0.409	n
Oik29a	0.131	14	0.183	14	0.294	13	0.259	0.224	n
Oik29b	0.161	20	0.194	20	0.385	4	0.226	0.211	n
Oik30a	0.373	20	0.271	20	0.247	13	0.195	0.230	n
Oik30b	0.263	20	0.267	20	0.333	12	0.267	0.267	n
Oik30c	0.386	20	0.291	20	0.305	13	0.218	0.252	n

Oik30d	0.386	20	0.290	20	0.302	13	0.216	0.250	n
Oik30e	0.386	20	0.290	20	0.301	13	0.216	0.250	n
Oik31a	0.187	23	0.302	23	0.755	3	0.488	0.402	n
Oik31b	0.169	25	0.272	17	0.625	3	0.510	0.401	n
Oik32	0.292	20	0.461	20	0.903	2	0.703	0.591	**Y**
Oik33a	0.144	14	0.230	14	0.616	13	0.352	0.296	n
Oik33b	0.140	20	0.267	14	0.750	13	0.508	0.397	n
Oik34a	0.118	17	0.184	4	0.371	3	0.341	0.269	n
Oik34b	0.118	17	0.184	4	0.371	3	0.340	0.268	n
Oik35	0.329	22	0.321	22	0.517	1	0.303	0.312	n
Oik36a	0.359	16	0.433	16	0.612	4	0.491	0.464	**Y**
Oik36b	0.359	16	0.433	16	0.612	4	0.491	0.464	**Y**
Oik37	0.482	35	0.309	35	0.415	32	0.172	0.235	n
Oik38	0.185	16	0.310	16	0.637	1	0.464	0.393	n
Oik39	0.114	15	0.146	15	0.474	13	0.206	0.178	n
Oik40a	0.300	20	0.289	20	0.388	1	0.262	0.275	n
Oik40b	0.186	20	0.207	20	0.320	1	0.217	0.213	n
Oik41a	0.245	20	0.247	20	0.382	1	0.229	0.237	n
Oik41b	0.245	20	0.247	20	0.382	1	0.229	0.237	n
Oik42	0.224	27	0.345	18	0.746	15	0.563	0.463	**Y**
Oik43	0.110	26	0.140	5	0.231	6	0.181	0.162	n
Oik44	0.155	26	0.235	14	0.489	1	0.393	0.320	n
Oik45	0.564	17	0.439	17	0.451	3	0.313	0.371	n
Oik46	0.140	20	0.202	17	0.444	4	0.312	0.262	n
Oik47	0.150	33	0.141	3	0.199	2	0.195	0.170	n
Oik48	0.312	15	0.473	15	0.778	6	0.723	0.608	**Y**
Oik49a	0.178	22	0.289	14	0.547	1	0.487	0.396	n
Oik49b	0.195	22	0.290	20	0.606	1	0.448	0.375	n
Oik50	0.193	19	0.272	19	0.500	5	0.387	0.334	n
Oik51a	0.134	14	0.272	14	0.668	13	0.548	0.421	n
Oik51b	0.145	14	0.277	14	0.678	13	0.558	0.429	n
Oik51c	0.140	20	0.204	20	0.401	3	0.295	0.253	n
Oik51d	0.140	20	0.204	20	0.401	3	0.295	0.253	n

Definitions of scores / positions can be found at http://www.cbs.dtu.dk/services/SignalP-4.0/output.php. A D-score above 0.450 predicts a signal peptide (**Y**) whereas a score below this threshold does not (n).
