# Supplementary material for: The Evolving Proteome of a Complex Extracellular Matrix, the Oikopleura House
Source: PLoS One. 2012 Jul 5;7(7):e40172. doi: 10.1371/journal.pone.0040172 (PMC3390340; doi:10.1371/journal.pone.0040172)
Supplement: Table S2 — SecretomeP 2.0 prediction of secretion of oikosins not predicted to have a signal peptide by SignalP 4.0. Non-classically secreted proteins should have an NN-score exceeding a threshold of 0.5. (PDF) [file pone.0040172.s007.pdf]

SUPPORTING TABLE S2

Table S2. SecretomeP 2.0 prediction of secretion of oikosins not predicted to have a signal peptide by SignalP 4.0.

Oikosin	NN-score	Secretion Prediction
Oik3	0.786	Y
Oik6a-e	0.538- 0.704	Y
Oik7	0.973	Y
Oik8	0.629	Y
Oik9	0.806	Y
Oik12	0.509	Y
Oik13	0.438	n
Oik15	0.908	Y
Oik16	0.768	Y
Oik17a	0.437	n
Oik17b	0.548	Y
Oik20	0.806	Y
Oik21a	0.358	n
Oik21b	0.251	n
Oik22	0.472	n
Oik23	0.423	n
Oik24b-g	0.523- 0.721	Y
Oik27	0.717	Y
Oik28a,b	0.622, 0.560	Y
Oik29a,b	0.565, 0.733	Y
Oik30a-d	0.501- 0.522	Y
Oik30e	0.458	n
Oik31a	0.688	Y
Oik31b	0.700	Y
Oik33a	0.438	n
Oik33b	0.500	Y
Oik34a	0.430	n
Oik34b	0.320	n
Oik35	0.464	n
Oik37	0.850	Y
Oik38	0.747	Y
Oik39	0.669	Y
Oik40a,b	0.539, 0.544	Y
Oik41a,b	0.627, 0.662	Y
Oik43	0.406	n
Oik44	0.649	Y
Oik45	0.873	Y
Oik46	0.830	Y
Oik47	0.589	Y
Oik49a	0.870	Y
Oik49b	0.851	Y
Oik50	0.735	Y
Oik51a,b	0.788, 0.749	Y
Oik51c,d	0.380, 0.410	Y

Non-classically secreted proteins should have an NN-score exceeding a threshold of 0.5.
