# Supplementary material for: The Evolving Proteome of a Complex Extracellular Matrix, the Oikopleura House
Source: PLoS One. 2012 Jul 5;7(7):e40172. doi: 10.1371/journal.pone.0040172 (PMC3390340; doi:10.1371/journal.pone.0040172)
Supplement: Table S3 — Primers generating in situ probes between 250 and 800 bp for newly identified oikosins. (PDF) [file pone.0040172.s008.pdf]

SUPPORTING TABLE S3

Table S3. Primers generating *in situ* probes between 250 and 800 bp for newly identified oikosins.

Oikosin	Forward Primer (5' to 3')	Reverse Primer (5' to 3')
Oik9	AGCTACAGCAGCGGCTACAACTAT	TTGACTTTGTATTCACCAGCGGCG
Oik10	AGCTGAAGAAGCAGCAGCAACAAG	TCCTCCAGGAAGAACGTGAGCATT
Oik11	AATGGGCAAATCTTTCCAGCAGGG	TCAGCGGTTTCGTCAACTCTGTCT
Oik12	ATTCGGCTCAACTTACACCTCCGT	TGCGGGTGGTAGTTGTTCTCGTTA
Oik13	TGTCAGTTCCACTGATGCCACCTT	TTCGAAAGTAGAGCTTGTGCCGGA
Oik14	AGAGCTTTGCTTTGCTTCCAAGGG	ACTTCGAAGCTGGCAGTAGCGTAA
Oik15	ATTTCGCTGGCTGCTCGACGTATT	TTGACCGACGCATTTGGCGTAATC
Oik16	TTCAAATGGCAGATGATGCGACCG	TCTGCGTGGTTTCGGTAGTAAGCA
Oik17a	TCAACGCTGACGGTACTGGTGGAA	ACGTTGGTAAACCGACGACCTTCT
Oik19	TCCCTGGCTCATACAACTGCTCTT	ACATTCAAACCCGTCACCGGAGTA
Oik20	CAACGTCGTCAACGCTGCTGATTT	ATGGCGAGACCAACGTCCTTGTAT
Oik21a	ACAAGAAACCCTGTCGCCTCGTAT	CCTGCAGATTTGAGTTTGCGCTGA
Oik22	CAACTTGCTGCCAAGCATCATGGA	AGCATAAGTGGCTTCTGAGCTGGA
Oik23	ACGCCACAATGATTGCTGTTGAGG	TCAGTGCACTCAGCGGTACAGAAA
Oik24a	TGGATCGATACTGGCCATCCCTTT	TACTCATCGCCGCAGTTGTGGTTA
Oik25	TCAATATGCCCGACTCTGATGGCA	GAGCCATCATCGTTGTTGGTGCAA
Oik26	TGCAAGTCGATACGCACGAGTTCT	CGCGAATCGCTTCTCGATCTTCTT
Oik27	TCGACGACTTGTCATCAACGAGCA	AGCTTCTTCATTCGTCGCACAACC
Oik28a	AGCTGTTTGCACATGGTTTCCGAC	TCGGCTTCTTCGAGAACTTCGGTT
Oik29a	TCGATGAGGCTGACAAGAACACCA	TCGCCCATCTCGCAAGAAAGTTTG
Oik30a	TGCTGACTATGAGCGAATCTGCGA	TTGATGCACCCACAGGAGTTCTCT
Oik31a	TGTCGAGATGGCACGAACTGATGA	TTGTCGCACATGAATCGGCATCTG
Oik33a	GCCTTTGTTGGACCAATCGACGTT	ATCGATTTCCTGGGTTTCCGTCCT
Oik34a	TCGACGACGACTTTGTCAACCTGT	CGTGCCCGTGTTGATATGCACAAT
Oik35	ACAGCGTTTACCACGGCTCTACTT	ATGTGGAATCGGAGAGTTGGTCGT
Oik36a	GCGCAACTTGCCCAAAGAACAAAG	TGATTTCCTTGGCTTGCCCATTCG
Oik37	ATACGACACAACACCGAGCTCACA	AGCTTGTCGTTCGCCCATTCTTTG
Oik38	AGAAGGATGCGCGATACGTGTCAA	TTGACCTTCTTGAGTCGTCGGACA
Oik39	TGTTATCCTCGGCGTTGTTGTTGC	TTGTCGACCCATCGGTTGTACATGG
Oik40a	AGCCGTGCTGACCCAGTACAATTT	ACTGAATCCAGTCGTCGAGCATGT
Oik41a	ACCACAACGACGACAACAACAACG	TCTGACCCATCAGCATGGACAACT
Oik42	TGTCGACGATAATGACGAGCCAGA	TTCATCAGAACCATCGACGGCGTA
Oik43	GCAATTGGGACCATTCTGCAACGA	TCGGGATTGGAAGCCTCGTTCTTA
Oik44	GAACAGGTTGCTTGCAACTGGGAA	TTGTCGTGGTAAGGGAATCGAGCA
Oik45	CTTTGGACTCTGCATTGGCTGCAT	AAAGCACATGCCATCAGTCGAACC
Oik46	TTCGAAAGCACCACCATTGGCTTC	TGCCTTCAAGCTCCTGGTATTGGA
Oik47	TGTCCAAAGGGCTTCTACCAGGTT	CGCAATGCCCAGTGACAAAGTCAT
Oik48	AGATGACGGCGTAGTCGCATACAA	TCTTCGACGGAAGTAGCTTCGCAA
Oik49a	AAGCTGCTGACTTCTACTCCGACA	CACTGCTTGAAGTCCTTGCACGAT
Oik50	ATTCCTGAGCGAACCAGCTTCTGA	GCTGAAGATCGCACTGGCAAATGT
Oik51a	ACCGAAGTTATCAGCGACCTTTCC	ACCATTTGCAGGAGTAGAGCCAGT
