# Supplementary material for: The Evolving Proteome of a Complex Extracellular Matrix, the Oikopleura House
Source: PLoS One. 2012 Jul 5;7(7):e40172. doi: 10.1371/journal.pone.0040172 (PMC3390340; doi:10.1371/journal.pone.0040172)
Supplement: Table S4 — BLASTp similarities of oikosin proteins to Ciona intestinalis and deuterostomes. Oik, oikosin; % cov, % coverage; e-val, BLASTp e-value; Non-deut e-val, lowest e-value from Table S5 for the given oikosin; -, no similarities found. (PDF) [file pone.0040172.s009.pdf]

SUPPORTING TABLE S4

Table S4. BLASTp similarities of oikosin proteins to *Ciona intestinalis* and deuterostomes.

Oik	MW (kDa)	BLASTp *Ciona*	% cov	e-val	% sim	BLASTp Swissprot	Deuterostome Organism	Deuterostome Class	% cov	e-val	% sim	Non-Deut e-val
1	256	EGF domain protein	56	1e-31	40	Oikosin-like protein	*Saccoglossus kowalevskii*	Enteropneusta	51	3e-60	49	-
2	84	-	-	-	-	-	-	-	-	-	-	-
3	67	-	-	-	-	-	-	-	-	-	-	-
4	32	Similar to fibrillin 1	27	1e-03	36	Hypothetical protein	*Branchiostoma floridae*	Cephalochordata	58	3e-08	58	2e-07
5	42	-	-	-	-	-	-	-	-	-	-	-
6a	51	EGF domain protein	12	6e-06	35	Similar to Cubilin	*Strongylocentrotus purpuratus*	Echinoidea	34	7e-09	45	5e-12
6b	53	EGF domain protein	13	9e-05	31	Cubilin-like	*Cavia porcellus*	Vertebrata	35	2e-07	38	3e-12
6c	55	EGF domain protein	12	3e-05	31	Cubn protein	*Danio rerio*	Vertebrata	28	4e-08	48	1e-11
6d	55	EGF domain protein	12	6e-07	34	Cubilin-like	*Cavia porcellus*	Vertebrata	30	1e-08	39	1e-10
6e	73	EGF domain protein	17	7e-07	38	Tolloid-like 2	*Tetraodon nigroviridis*	Vertebrata	53	2e-13	40	4e-12
7	21	-	-	-	-	-	-	-	-	-	-	-
8	36	-	-	-	-	-	-	-	-	-	-	-
9	71	-	-	-	-	-	-	-	-	-	-	-
10	57	-	-	-	-	-	-	-	-	-	-	-
11	39	-	-	-	-	-	-	-	-	-	-	-
12	35	-	-	-	-	-	-	-	-	-	-	-
13	54	sim. to superoxide-dismutase	53	3e-15	50	Cu-Zn Superoxide Dismutase	*Saccoglossus kowalevskii*	Enteropneusta	58	1e-19	46	8e-21
14	482	Similar to polycystic kidney and hepatic disease 1	74	7e-171	46	Fibrocystin-L	*Danio rerio*	Vertebrata	77	0.0	44	0.0
15	26	-	-	-	-	-	-	-	-	-	-	-
16	21	-	-	-	-	-	-	-	-	-	-	-
17a	76	-	-	-	-	-	-	-	-	-	-	1e-03
17b	68	-	-	-	-	-	-	-	-	-	-	-
18	27	-	-	-	-	-	-	-	-	-	-	-
19	153	EGF domain protein	72	6e-153	51	EGF domain protein	*Branchiostoma floridae*	Cephalochordata	69	0.0	54	8e-154
20	42	-	-	-	-	CUB and zona pellucida-like domain containing protein	*Danio rerio*	Vertebrata	64	2e-3	39	-
21a	94	sim. to oxidase/peroxidase	71	3e-76	48	similar to oxidase/peroxidase	*Branchiostoma floridae*	Cephalochordata	69	2e-74	47	1e-83
21b	87	sim. to oxidase/peroxidase	69	6e-73	47	similar to oxidase/peroxidase	*Branchiostoma floridae*	Cephalochordata	70	2e-63	48	5e-70

22	33	-	-	-	-	-	-	-	-	-	-	-
23	244	sim. to HyTSR1 protein	4	7e-10	52	Hemicentin-1	*Heterocephalus glaber*	Vertebrata	10	6e-08	41	2e-08
24a	70	Zn-dependent metalloprotease, astacin_like	37	1e-10	41	astacin-like metallo-endopeptidase	*Xenopus laevis*	Vertebrata	19	6e-10	48	2e-08
24b	83	Similar to fibrillin 2	16	1e-05	42	astacin-like metallo-endopeptidase	*Xenopus laevis*	Vertebrata	20	3e-09	45	2e-09
24c	85	Sim. to microneme protein 4	15	1e-05	48	-	-	-	-	-	-	3e-08
24d	92	MEP1A protein	6	3e-05	45	Hypothetical protein	*Ailuropoda melanoleuca*	Vertebrata	13	2e-12	58	2e-19
24e	78	Zn-dependent metalloprotease	15	1e-05	34	astacin-like metallo-endopeptidase	*Xenopus laevis*	Vertebrata	21	2e-08	45	3e-08
24f	80	Predicted protein	26	5e-09	44	astacin-like metallo-endopeptidase	*Xenopus tropicalis*	Vertebrata	21	6e-09	47	3e-10
24g	149	Serine protease	19	2e-16	26	Trypsin-like serine protease	*Xenopus laevis*	Vertebrata	49	4e-43	42	2e-33
24h	172	Serine protease	16	3e-14	28	Ovochymase-1-like	*Xenopus tropicalis*	Vertebrata	48	4e-47	38	-
25	68	Calcium-binding EGF-like domain	72	2e-35	44	Calcium-binding EGF-like domaim	*Branchiostoma floridae*	Cephalochordata	77	5e-53	46	2e-30
26	16	-	-	-	-	-	-	-	-	-	-	-
27	16	-	-	-	-	-	-	-	-	-	-	-
28a	71	sim. to fibrillin 1	22	2e-11	55	similar to microneme protein 4	*Strongylocentrotus purpuratus*	Echinoidea	26	1e-11	56	6e-12
28b	89	sim. to fibrillin 1	31	1e-51	59	Calcium-binding EGF-like domain	*Branchiostoma floridae*	Cephalochordata	47	2e-43	49	3e-47
29a	38	Galactose binding lectin domain	31	1e-07	50	plasminogen-like	*Saccoglossus kowalevskii*	Enteropneusta	39	4e-08	51	5e-07
29b	37	Galactose binding lectin domain	27	2e-07	55	glycerol-3-phosphate dehydrogenase 2	*Saccoglossus kowalevskii*	Enteropneusta	26	9e-07	52	6e-08
30a	254	sim. to Fc fragment of IgG bind. protein	73	3e-42	36	IgGFc-binding protein-like	*Anolis carolinensis*	Vertebrata	74	8e-81	37	-
30b	276	EGF domain protein	3	4e-06	36	IgGFc-binding protein-like	*Xenopus tropicalis*	Vertebrata	68	3e-80	39	-
30c	277	sim. to Fc fragment of IgG bind. protein	63	6e-48	37	IgGFc-binding protein-like	*Xenopus tropicalis*	Vertebrata	67	2e-77	38	-
30d	256	sim. to Fc fragment of IgG bind. protein	72	3e-41	38	IgGFc-binding protein-like	*Xenopus tropicalis*	Vertebrata	71	2e-83	39	-
30e	400	EGF domain protein	2	1e-06	38	IgGFc-binding protein-like	*Xenopus tropicalis*	Vertebrata	60	9e-80	38	-
31a	25	-	-	-	-	-	-	-	-	-	-	-
31b	26	-	-	-	-	-	-	-	-	-	-	-
32	55	sim. to fibrillin 2	70	2e-18	45	Calcium-binding EGF-like domain	*Branchiostoma floridae*	Cephalochordata	65	3e-25	44	5e-17
33a	176	-	-	-	-	-	-	-	-	-	-	1e-15
33b	175	-	-	-	-	-	-	-	-	-	-	3e-20
34a	135	-	-	-	-	-	-	-	-	-	-	8e-29
34b	132	-	-	-	-	-	-	-	-	-	-	2e-27
35	57	sim. to HyTSR1 protein	48	2e-30	53	Matrilin-like	*Xenopus laevis*	Vertebrata	37	1e-25	53	-
36a	33	Gal_lectin binding domain	64	4e-21	48	L-rhamnose-binding lectin CSL1-like	*Oreochromis niloticus*	Vertebrata	69	7e-23	49	5e-22
36b	33	Gal_lectin binding domain	64	2e-20	47	L-rhamnose-binding lectin CSL1-like	*Oreochromis niloticus*	Vertebrata	69	8e-23	49	4e-22

37	34	-	-	-	-	-	-	-	-	-	-	-
38	15	-	-	-	-	-	-	-	-	-	-	-
39	30	-	-	-	-	-	-	-	-	-	-	-
40a	120	-	-	-	-	-	-	-	-	-	-	-
40b	120	-	-	-	-	-	-	-	-	-	-	-
41a	198	-	-	-	-	-	-	-	-	-	-	-
41b	199	-	-	-	-	-	-	-	-	-	-	-
42	93	-	-	-	-	C-type lectin, Calcium-binding EGF-like domain	*Branchiostoma floridae*	Cephalochordata	49	2e-06	35	-
43	158	EGF domain protein	73	2e-38	38	C-type lectin, Calcium-binding EGF-like domain	*Branchiostoma floridae*	Cephalochordata	68	3e-56	41	4e-45
44	27	-	-	-	-	-	-	-	-	-	-	-
45	40	-	-	-	-	-	-	-	-	-	-	-
46	51	-	-	-	-	-	-	-	-	-	-	-
47	165	Similar to zonadhesin	28	1e-03	24	granulinin	*Saccoglossus kowalevskii*	Enteropneusta	29	2e-10	32	1e-13
48	29	-	-	-	-	-	-	-	-	-	-	-
49a	29	-	-	-	-	-	-	-	-	-	-	-
49b	30	-	-	-	-	-	-	-	-	-	-	-
50	30	-	-	-	-	-	-	-	-	-	-	-
51a	66	-	-	-	-	C-lect	*Branchiostoma floridae*	Cephalochordata	16	3e-05	47	-
51b	67		17	3e-05	45	C-lect	*Branchiostoma floridae*	Cephalochordata	16	5e-03	59	-
51c	86	-	-	-	-	-	-	-	-	-	-	-
51d	86	-	-	-	-	-	-	-	-	-	-	-

Oik, oikosin; % cov, % coverage; e-val, BLASTp e-value; Non-deut e-val, lowest e-value from **Table S5** for the given oikosin; -, no similarities found.
