# Supplementary material for: The Evolving Proteome of a Complex Extracellular Matrix, the Oikopleura House
Source: PLoS One. 2012 Jul 5;7(7):e40172. doi: 10.1371/journal.pone.0040172 (PMC3390340; doi:10.1371/journal.pone.0040172)
Supplement: Table S5 — BLASTp similarities of oikosin proteins to non-deuterostome organisms. Oik, oikosin; % cov, % coverage; e-val, BLASTp e-value; -, no similarities found.for the given oikosin; -, no similarities found. (PDF) [file pone.0040172.s010.pdf]

SUPPORTING TABLE S5

Table S5. BLASTp similarities of oikosin proteins to non-deuterostome organisms.

Oik	MW (kDa)	Non-deutersostome protein	Non-deuterostome organism	Class	% cov	e-val	% sim
1	256	-	-	-	-	-	-
2	84	-	-	-	-	-	-
3	67	-	-	-	-	-	-
4	32	Cubilin	*Camponotus floridans*	Insecta	48	2e-07	50
5	42	-	-	-	-	-	-
6a	51	Hypothetical protein	*Nematostella vectensis*	Anthozoa	62	5e-12	37
6b	53	Predicted protein	*Nematostella vectensis*	Anthozoa	60	3e-12	38
6c	55	Predicted protein	*Nematostella vectensis*	Anthozoa	58	1e-11	38
6d	55	Predicted protein	*Nematostella vectensis*	Anthozoa	58	1e-10	37
6e	73	Hypothetical protein	*Ixodes scapularis*	Arachnida	41	4e-12	36
7	21	-	-	-	-	-	-
8	36	-	-	-	-	-	-
9	71	-	-	-	-	-	-
10	57	-	-	-	-	-	-
11	39	-	-	-	-	-	-
12	35	-	-	-	-	-	-
13	54	Cu-Zn Superoxide Dismutase	*Populus trichocarpa*	Eudicotyledons	50	8e-21	64
14	482	Fibrocystin-L	*Amphimedon queenslandica*	Demospongiae	80	0.0	43
15	26	-	-	-	-	-	-
16	21	-	-	-	-	-	-
17a	76	lipoprotein	*Desulfotomaculum kuznetsovii*	Clostridia	11	1e-03	46
17b	68	-	-	-	-	-	-
18	27	-	-	-	-	-	-
19	153	EGF domain protein	*Trichoplax adhaerens*	Trichoplacoidea	71	8e-154	56
20	42	-	-	-	-	-	-
21a	94	Chorion peroxidase	*Acromyrmex echinatior*	Insecta	70	1e-83	50
21b	87	Peroxidase-like	*Daphnia pulex*	Branchiopoda	70	5e-70	48
22	33	-	-	-	-	-	-

23	244	Hemicentin-1	*Ascaris suum*	Chromadorea	14	2e-08	35
24A	70	Zinc-dependent metalloprotease, astacin_like	*Nematostella vectensis*	Anthozoa	17	2e-08	48
24b	83	Zinc-dependent metalloprotease, astacin_like	*Anopheles gambiae*	Insecta	17	2e-09	50
24c	85	Zinc-dependent metalloprotease, astacin_like	*Acyrthosiphon pisum*	Insecta	14	3e-08	50
24d	92	LPXTG-motif cell wall anchor domain protein	*Lactobacillus reuteri*	Bacilli	13	2e-19	60
24e	78	metalloproteinase	*Aedes aegypti*	Insecta	18	3e-08	45
24f	80	metalloproteinase	*Culex quinquefasciatus*	Insecta	17	3e-10	50
24g	149	Trypsin-like serine protease	*Trichoplax adhaerens*	Trichoplacoidea	44	2e-33	47
24h	172	-	-	-	-	-	-
25	68	Calcium-binding EGF-like domain	*Nematostella vectensis*	Anthozoa	70	2e-30	47
26	16	-	-	-	-	-	-
27	16	-	-	-	-	-	-
28a	71	Predicted protein	*Nematostella vectensis*	Anthozoa	13	6e-12	56
28b	89	Calcium-binding EGF-like domain	*Salpingoeca sp*	Salpingoecidae	44	3e-47	55
29a	38	Galactose binding lectin domain	*Daphnia pulex*	Branchiopoda	31	5e-07	54
29b		Galactose binding lectin domain	*Nematostella vectensis*	Anthozoa	30	6e-08	53
30a	253	-	-	-	-	-	-
30b	276	-	-	-	-	-	-
30c	277	-	-	-	-	-	-
30d	256	-	-	-	-	-	-
30e	400	-	-	-	-	-	-
31a	25	-	-	-	-	-	-
31b	26	-	-	-	-	-	-
32	55	Calcium-binding EGF-like domain	*Trichoplax adhaerens*	Trichoplacoidea	59	5e-17	44
33a	176	similar to tyrosine kinase receptor	*Hydra magnipapillata*	Hydrozoa	73	1e-15	40
33b	175	similar to tyrosine kinase receptor	*Hydra magnipapillata*	Hydrozoa	78	3e-20	42
34a	135	similar to tyrosine kinase receptor	*Hydra magnipapillata*	Hydrozoa	72	8e-29	41
34b	132	similar to tyrosine kinase receptor	*Hydra magnipapillata*	Hydrozoa	72	2e-27	43
35	57	-	-	-	-	-	-
36a	33	Galactose binding lectin domain	*Hydra magnipapillata*	Hydrozoa	75	5e-22	48
36b	33	Galactose binding lectin domain	*Hydra magnipapillata*	Hydrozoa	75	4e-22	50
37	34	-	-	-	-	-	-
38	15	-	-	-	-	-	-

39	30	-	-	-	-	-	-
40a	120	-	-	-	-	-	-
40b	120	-	-	-	-	-	-
41a	198	-	-	-	-	-	-
41b	199	-	-	-	-	-	-
42	93	-	-	-	-	-	-
43	158	Calcium-binding EGF-like domain	*Monosiga brevicollis*	Opisthokonta	71	4e-45	38
44	27	-	-	-	-	-	-
45	40	-	-	-	-	-	-
46	51	-	-	-	-	-	-
47	165	Predicted protein	*Trichoderma reesei*	Sordariomycetes	88	1e-13	37
48	29	-	-	-	-	-	-
49a	29	-	-	-	-	-	-
49b	30	-	-	-	-	-	-
50	30	-	-	-	-	-	-
51a	66	-	-	-	-	-	-
51b	67	-	-	-	-	-	-
51c	86	-	-	-	-	-	-
51d	86	-	-	-	-	-	-

Oik, oikosin; % cov, % coverage; e-val, BLASTp e-value; -, no similarities found.
