# Supplementary material for: The Evolving Proteome of a Complex Extracellular Matrix, the Oikopleura House
Source: PLoS One. 2012 Jul 5;7(7):e40172. doi: 10.1371/journal.pone.0040172 (PMC3390340; doi:10.1371/journal.pone.0040172)
Supplement: Data S1 — Accession numbers for oikosins. (PDF) [file pone.0040172.s011.pdf]

SUPPORTING DATA S1

Supporting Data S1. Accession numbers for oikosins: 1 (AJ308491), 2 (AJ308492), 3 (AJ308495), 4 (AJ310624), 5 (AJ310627), 6 (AJ310629), 7 (AJ310634), 8 (FN806849), 9 (HE663406), 10 (HE663407), 11 (HE663408), 12 (HE663409), 13 (HE663410), 14 (HE663411), 15 (HE663412), 16 (HE774605), 17a (HE774606), 17b (HE774607), 18 (FN806850), 19 (HE774608), 20 (HE774609), 21a (HE774610), 21b (HE774611), 22 (HE774612), 23 (HE774613), 24a (HE774614), 24b (HE774615), 24c (HE774616), 24d (HE774617), 24e (HE774618), 24f (HE774619), 24g (HE774620), 24h (HE774621), 25 (HE774622), 26 (HE774623), 27 (HE774624), 28a (HE774625), 28b (HE774626), 29a (HE774627), 29b (HE774628), 30a (HE774629), 30b (HE774630), 30c (HE774631), 30d (HE774632), 30e (HE774633), 31a (HE774634), 31b (HE774635), 32 (FN806851), 33a (HE774636), 33b (HE774637), 34a (HE774638), 34b (HE774639), 35 (HE774640), 36a (HE774641), 36b (HE774642), 37 (HE774643), 38 (HE774644), 39 (HE774645), 40a (HE774645), 40b (HE774645), 41a (HE774645), 41b (HE774645), 42 (HE774645), 43 (HE774645), 44 (HE774645), 45 (HE774645), 46 (HE774645), 47 (HE774645), 48 (HE774645), 49a (HE774645), 49b (HE774645), 50 (HE774645), 51a (HE774645), 51b (HE774645), 51c (HE774645), and 51d (HE774645).
